# Navigating interfacial challenges in lithium metal batteries: from fundamental understanding to practical realization

**DOI:** 10.1186/s40580-025-00491-4

**Published:** 2025-05-29

**Authors:** Jimin Lee, Youngbin Park, Jang Wook Choi

**Affiliations:** School of Chemical and Biological Engineering, Institute of Chemical Process, Seoul National University, 1 Gwanak-ro, Gwanak-gu, Seoul, 08826 Republic of Korea

**Keywords:** Corrosion, Dendrites, Electrolyte engineering, Lithium metal batteries, Solid-electrolyte interphase, Surface modification

## Abstract

Lithium metal batteries (LMBs) hold immense potential as next-generation energy storage systems due to their exceptionally high theoretical energy density. However, their commercialization is hindered by persistent interfacial instabilities that accelerate capacity degradation and limit cycle life. A major challenge lies in the solid-electrolyte interphase (SEI), whose composition and structure critically influence lithium deposition behavior, electrolyte stability, and overall battery performance. This review examines key aspects of SEI stability and its impact on battery performance, highlighting recent advancements in electrolyte engineering and surface modification strategies aimed at enhancing interfacial stability. Beyond laboratory-scale optimizations, we discuss key considerations for translating these advancements into industrial applications, highlighting the importance of practical testing protocols to bridge the gap between fundamental research and real-world deployment.

## Graphical Abstract

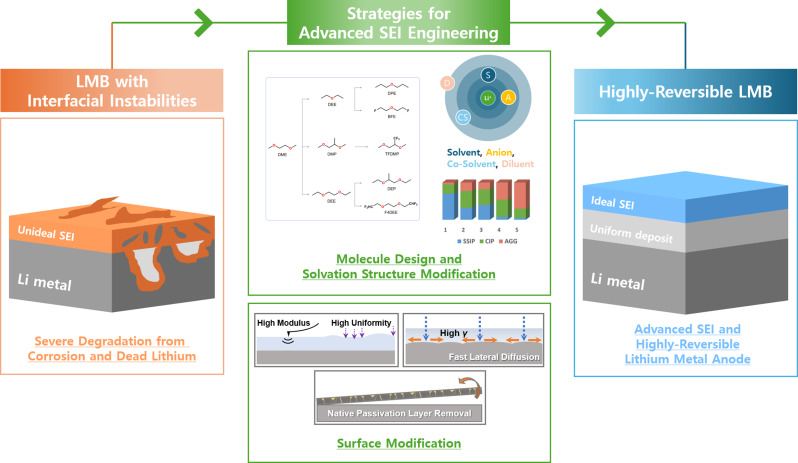

## Introduction: the transition beyond conventional LIBs

Since their commercialization in the 1990s, lithium-ion batteries (LIBs) have become the industry standard for rechargeable energy storage, powering applications from portable electronics to electric vehicles and grid-scale energy systems [1–3]. The widespread adoption of LIBs is attributed to their relatively high energy density, electrochemical reversibility, and scalable manufacturing. However, as energy storage demands continue to grow, the inherent limitations of conventional LIBs, particularly the theoretical capacity constraints, are becoming increasingly evident.

In response, extensive research has been devoted to pushing the performance boundaries of existing LIB technology while maintaining its fundamental chemistry. Strategies such as integrating high-nickel layered oxide cathodes (e.g., Ni-rich NMC and NCA) to improve energy density [4, 5] and adopting lithium iron phosphate (LFP) cathodes for enhanced stability and cost reduction have been widely explored [6, 7]. While these approaches have led to incremental improvements, they remain fundamentally restricted by the intrinsic energy density limit imposed by the theoretical capacity of intercalation-based anodes, prompting the search for alternative materials that can offer a more substantial increase in energy density. One promising approach has been the development of silicon-based anodes [8, 9], which provide a theoretical specific capacity of ~ 3,600 mAh g^–1^, nearly ten times that of graphite (372 mAh g^–1^). While this dramatic increase in capacity suggests significant potential for improving LIB energy density, severe volumetric expansion (~ 300%) during cycling leads to structural degradation, loss of electrical contact, and rapid capacity fading. Addressing these issues requires advanced electrode engineering, such as nanoscale structuring, composite materials, and pre-lithiation strategies to improve cycle life and mechanical stability. Despite ongoing progress, the challenges associated with maintaining structural integrity and long-term reversibility continue to limit the practicality of silicon anodes.

A more fundamental shift in battery chemistry involves replacing intercalation-based anodes with lithium metal, which offers an exceptionally high theoretical specific capacity (3,860 mAh g^–1^) and lowest electrochemical potential (–3.04 V vs. SHE). These properties enable significantly higher energy densities compared to conventional LIBs, making lithium metal batteries (LMBs) one of the most promising candidates for next-generation energy storage technologies [10–12]. However, despite their theoretical advantages, LMBs face persistent challenges related to inherent electrochemical behavior [13]. Unlike graphite anodes, which relies on intercalation and de-intercalation mechanism, lithium metal undergoes deposition and stripping during charge and discharge cycles. While this mechanism allows for significantly higher capacity, it also increases the risk of dendritic growth, which can potentially cause short circuits and severe safety hazards. Additionally, the extremely low reduction potential of lithium makes it highly reactive, leading to continuous electrolyte decomposition even in the absence of an external voltage.

Although next-generation battery systems hold immense potential to revolutionize energy storage, their commercialization remains hindered by fundamental challenges, particularly those related to reversibility and long-term stability. In this regard, lithium metal batteries have shown substantial progress, as evidenced by recent reports of high Coulombic efficiencies (CE) exceeding 99.5%, highlighting their feasibility for practical applications [14]. Moreover, recent studies have demonstrated exceptionally high energy densities in LMBs, further underscoring their potential to meet the stringent demands of advanced energy storage systems. The concurrent advancements in both Coulombic efficiency and energy density have strengthened the feasibility of LMBs for practical deployment, ultimately reinforcing their promise as a next-generation energy storage solution.

Given this context, this review explores the remaining challenges in achieving the commercial feasibility of lithium metal batteries. By referencing recent milestone research, we consolidate current understanding on the degradation behavior of lithium metal anodes and outline related research trends. Recognizing that these degradation mechanisms are largely governed by the properties of the solid electrolyte interphase (SEI), we examine its compositional and structural aspects. We review the significant influence of organic and inorganic components on SEI properties and emphasize the importance of designing an SEI with an optimized composition and structure to improve battery performance and longevity.

## Remaining challenges in LMBs: current understanding and research efforts to address them

Achieving a Coulombic efficiency exceeding 99% signifies substantial technological advancements in lithium metal battery systems. However, degradation of lithium metal anodes—primarily driven by corrosion and the formation of dead lithium—remains a major challenge that undermines performance and reduces battery lifespan. ​In the following sections, we will explore these degradation phenomena incorporating the current state of understanding, and highlight research efforts aimed at mitigating these challenges.

### Chemical corrosion

Chemical corrosion refers to the spontaneous reactions occurring between lithium metal and the electrolyte. This process is particularly pronounced during calendar aging rather than continuous charge-discharge cycles. Although research on LMB calendar aging remains relatively modest, the extensive work on LIB calendar aging [15–17] can offer valuable insights, given the similarities in their system architectures. Across many experimental setups and battery types, the evidence is clear that higher state-of-charge (SoC) levels can accelerate the calendar aging of LIBs​. In the high SoC regime, the graphite anode reaches lower potentials, which accelerates the spontaneous degradation of electrolytes.

**Fig. 1:**
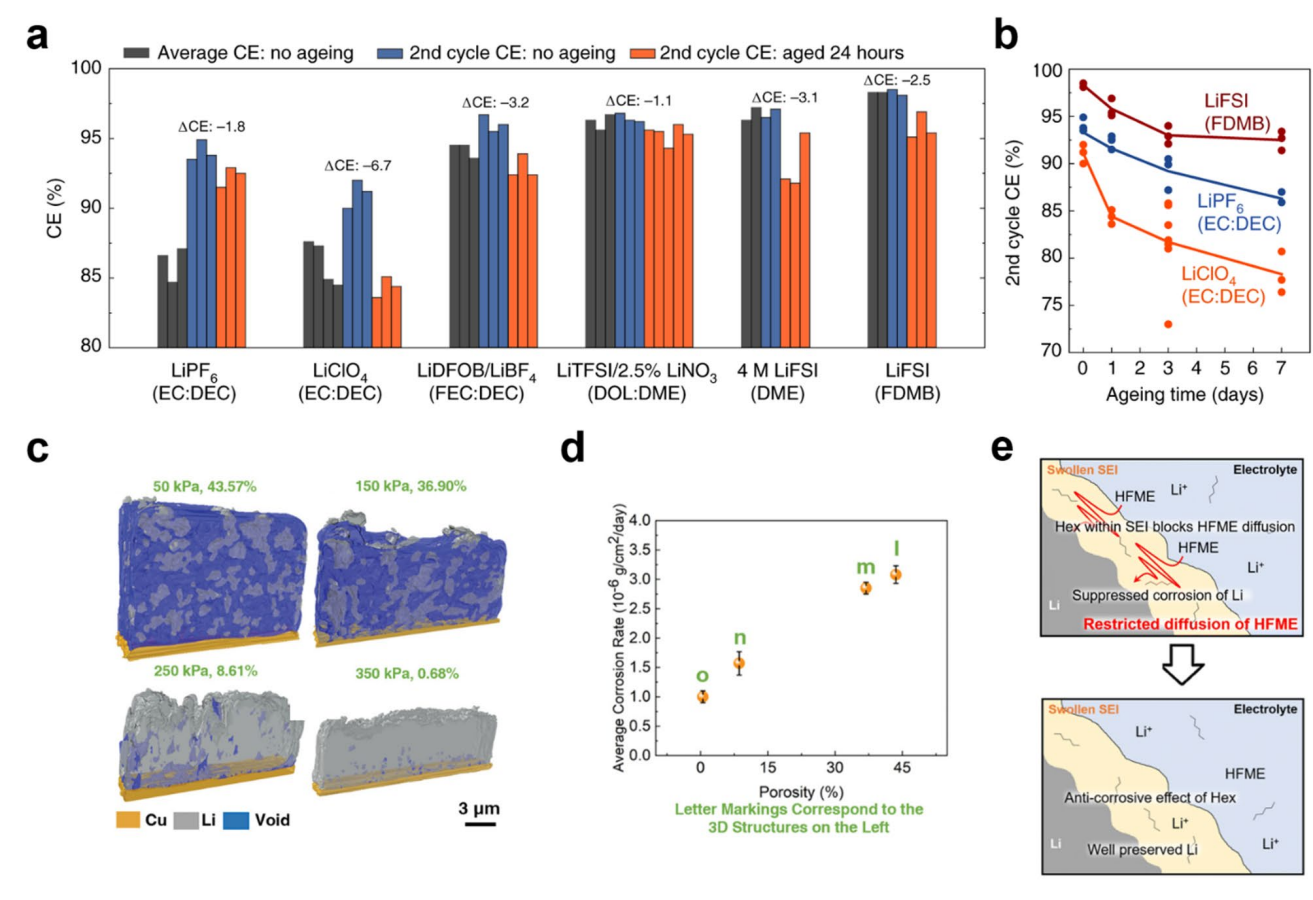
**a**) Coulombic efficiency of various electrolyte measured at the first and second cycle with and without aging. **b**) Reduction in Coulombic efficiency due to corrosion during aging. Reproduced with permission from Ref. [18]. Copyright 2021, Springer Nature. **c**) Applied stack pressure and the resulting lithium deposit structural changes with various porosities. **d**) Positive correlation between porosity and average corrosion rate. Reproduced with permission from Ref. [20]. Copyright 2022, John Wiley & Sons, Inc. **e**) Schematic diagram illustrating the anti-corrosive effect of n-hexane in LHCE electrolyte. Reproduced with permission from Ref. [21]. Copyright 2024, Royal Society of Chemistry

In this context, lithium metal anodes present a distinctive concern due to their inherently low reduction potential. This attribute leads lithium metal anodes to remain at low potential regardless of the SoC, and thus could predispose the anodes to continuous electrolyte decomposition. Recent research by Boyle et al. elucidates that, during rest periods, lithium metal undergoes spontaneous and persistent electrolyte decomposition—a corrosion process—leading to the progressive formation of the solid-electrolyte interphase on the lithium metal surface [18]. Their study demonstrates that lithium metal anodes undergo a capacity loss of at least 2–3% within 24 h of aging across the selected electrolyte compositions, which include not only conventional carbonate-based electrolytes (LiPF₆ in EC: DEC), but also high Coulombic efficiency electrolytes employing bisalt, electrolyte additives, high salt concentrations, and fluorinated solvents (Fig. 1a). Notably, certain electrolytes exhibiting CE above 95% still undergo substantial side reactions during aging, challenging a common assumption that high-CE electrolytes inherently form highly passivating SEI layers that mitigate electrolyte degradation (Fig. 1b). The authors explain that high-CE electrolytes offer advantages in controlling the morphology of electrodeposits, leading to higher CE. However, they argue that these electrolytes do not necessarily impede SEI growth or suppress side reactions over extended storage. Based on these findings, the authors emphasize the necessity of designing electrolytes that simultaneously minimize both the surface area of lithium deposits and the rate of electrolyte-induced corrosion per unit area. A recent study on the calendar life of lithium metal batteries corroborates these insights, indicating that minimizing the exposed lithium surface area to the electrolyte improves long-term capacity retention [19].

Lu et al. demonstrated that increasing stack pressure can effectively reduce the exposed surface area of lithium electrodeposits by decreasing their porosity, leading to a denser lithium deposit (Fig. 1c)​ ​ [20]. The porosity of electrodeposited lithium exhibits direct and positive correlation with the corrosion rate (Fig. 1d). By employing a localized high-concentration electrolyte (LHCE) and applying a stack pressure of 350 kPa, corrosion-induced lithium metal loss was minimized to approximately 0.8% over a 10-day immersion period. This finding highlights a viable strategy to mitigate corrosion-induced degradation without solely relying on complex electrolyte formulations. However, uniformly applying high stack pressure across the entire electrode surface in typical battery formats—such as cylindrical, prismatic, and pouch cells— may introduce additional technical challenges, potentially increasing costs and reducing system energy density. Additionally, significant corrosion persists at temperatures above 40 °C, underscoring the continued necessity of electrolyte modifications to further suppress degradation.

A recent study proposed enhancing corrosion resistance in localized high-concentration electrolytes by incorporating electrochemically inactive species [21]. Specifically, n-hexane was introduced into an LHCE with hexafluoroisopropyl methyl ether (HFME) as a diluent to mitigate lithium metal corrosion. The addition of n-hexane effectively suppressed lithium metal corrosion by acting as a kinetic barrier, preventing HFME from diffusing to the lithium surface through the swollen solid-electrolyte interphase (Fig. 1e). To assess corrosion mitigation and calendar life performance, electrochemical evaluation was conducted with an intentional 8-hour rest period after each charging process. Electrochemical testing demonstrated that a Li||NCM811 full-cell containing n-hexane retained 80.8% of its capacity after 160 cycles, whereas its hexane-free counterpart exhibited only 6.0% retention after 46 cycles, highlighting the effectiveness of this strategy in extending calendar life. Furthermore, immersion tests confirmed that n-hexane significantly reduced the formation of byproducts associated with HFME decomposition, reinforcing its role in mitigating chemical degradation. This approach demonstrates the potential of incorporating inert hydrocarbon diluents to improve both the cycle and calendar life of lithium metal batteries.

Another critical approach focuses on reinforcing the solid-electrolyte interphase itself. Since the SEI serves as the primary barrier between lithium metal and the electrolyte, its stability and structure play a pivotal role in determining the extent of lithium corrosion and overall battery longevity. Given its importance, recent research has focused on SEI engineering as a strategy to enhance lithium metal corrosion resistance. One study introduced a borate–pyran-based electrolyte to address persistent lithium corrosion issues​ [22]. This electrolyte restructures large LiF crystallites within the SEI into finely distributed crystalline or glassy LiF, improving interfacial passivity and significantly reducing electrolyte permeation. By effectively suppressing lithium corrosion, this approach minimizes electrolyte consumption, enabling lithium metal batteries to operate under lean-electrolyte conditions. As a result, LMBs utilizing this electrolyte achieved an initial energy density exceeding 400 Wh kg^–1^ and sustained 400 cycles with 70% capacity retention at an electrolyte-to-capacity (E/C) ratio of 1.92 g Ah^–1^, surpassing conventional lean-electrolyte systems where excessive electrolyte depletion typically limits longevity.

Another study tackled lithium corrosion by introducing an artificial passivation layer composed of a low-solubility polymer and embedded metal fluoride [23]. This layer inhibits SEI dissolution, reducing lithium metal exposure to the electrolyte and thereby minimizing continuous side reactions. Electrochemical testing demonstrated that the corrosion rate was reduced by approximately 74%, leading to prolonged cycling stability. These findings highlight promising strategies for mitigating lithium corrosion through electrolyte and interphase engineering, facilitating the development of longer-lasting lithium metal batteries.

### Galvanic corrosion

**Fig. 2:**
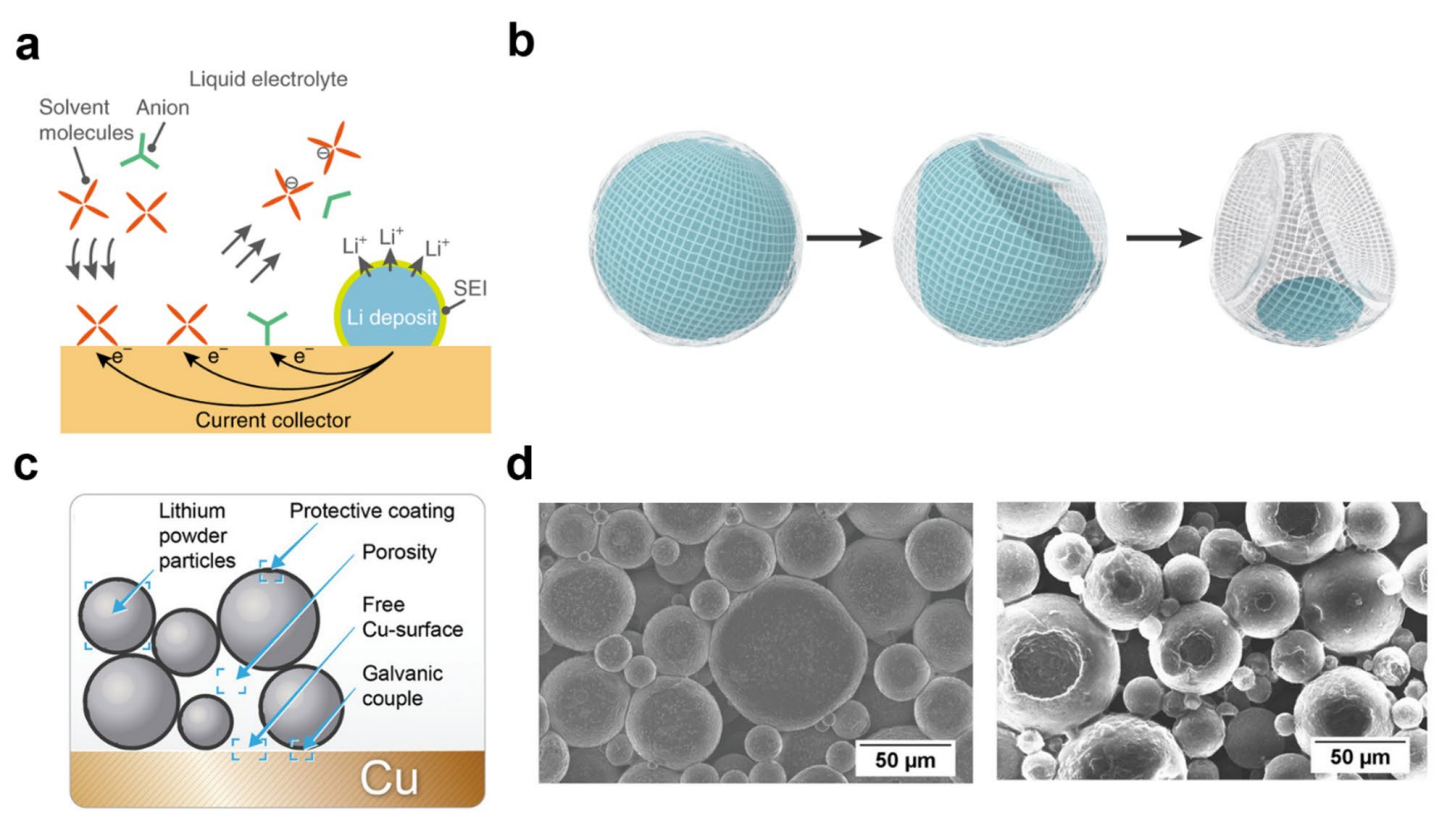
**a**) Schematic diagram illustrating the galvanic corrosion phenomena in lithium metal battery system. **b**) Schematic diagram illustrating the progression of Kirkendall-type corrosion. Reproduced with permission from Ref. [24]. Copyright 2019, Springer Nature. **c**) Schematic illustration of Li_p_ electrode on copper current collector. **d**) SEM images depicting the pitting corrosion of Li_p_ electrode. Reproduced with permission from Ref. [25], CC BY

Galvanic corrosion is an electrochemical degradation process that occurs when two dissimilar metals form a galvanic cell by being electrically connected in a shared electrolyte. In lithium metal batteries, this phenomenon manifests as a specific case where the lithium metal anode and copper current collector create a localized galvanic cell, driving unwanted side reactions. During this process, lithium oxidizes, but instead of direct electron transfer within the lithium metal, electrons migrate to copper, shifting electrolyte reduction reactions onto the copper surface (Fig. 2a).

A notable study provided new insights into the mechanism of galvanic corrosion in lithium metal batteries, showing that it can be described by a Kirkendall-type mechanism [24]​. This mechanism leads to void formation within lithium deposits, driven by disparities in inward and outward mass transport, a phenomenon characteristic of the Kirkendall effect (Fig. 2b). Morphological analysis using scanning electron microscopy (SEM) and cryogenic electron microscopy (cryo-EM) revealed that lithium deposits undergo rapid void formation, losing approximately 45.6% of their volume within 100 h of rest and nearly complete lithium depletion after 200 h. Electrochemical quantification further demonstrated that up to 80% of total lithium corrosion can be attributed to the galvanic process, emphasizing its significant role in battery degradation. Moreover, the study highlighted how this accelerated lithium loss can lead to structural instability, altered deposition behavior, and increased dendritic growth, ultimately compromising long-term battery performance.

In lithium metal batteries, galvanic corrosion occurs through decomposition of electrolyte components on the copper current collector. Thus, this effect is expected to be more pronounced in systems like anode-free LMB, where a large-area current collector is directly exposed to the electrolyte. Representatively, a study employing lithium-powder-based (Li_p_) electrodes demonstrated the severity of galvanic corrosion and its detrimental impact on lithium metal battery performance​ [25]. Li_p_ electrodes were chosen over conventional lithium foils due to their potential advantages. Their lower local current densities help suppress dendrite growth, while their porous structure accommodates volume changes, reducing mechanical stress and electrode degradation during cycling. However, despite these advantages, the study revealed that the porous structure of Li_p_-electrodes also exacerbates galvanic corrosion by increasing the exposure of the copper current collector to the electrolyte, facilitating the formation of a localized Cu||Li galvanic couple (Fig. 2c). For aged electrodes, severe pitting corrosion is also observed (Fig. 2d). Electrochemical impedance spectroscopy (EIS) and zero resistance ammetry (ZRA) measurements confirmed that galvanic corrosion increased overvoltage, negatively impacting electrode kinetics. Moreover, full-cell cycling tests with NMC 622 cathodes revealed rapid capacity degradation when aged Li_p_-electrodes were used, with performance deteriorating after only a few cycles.

### Dead lithium

**Fig. 3:**
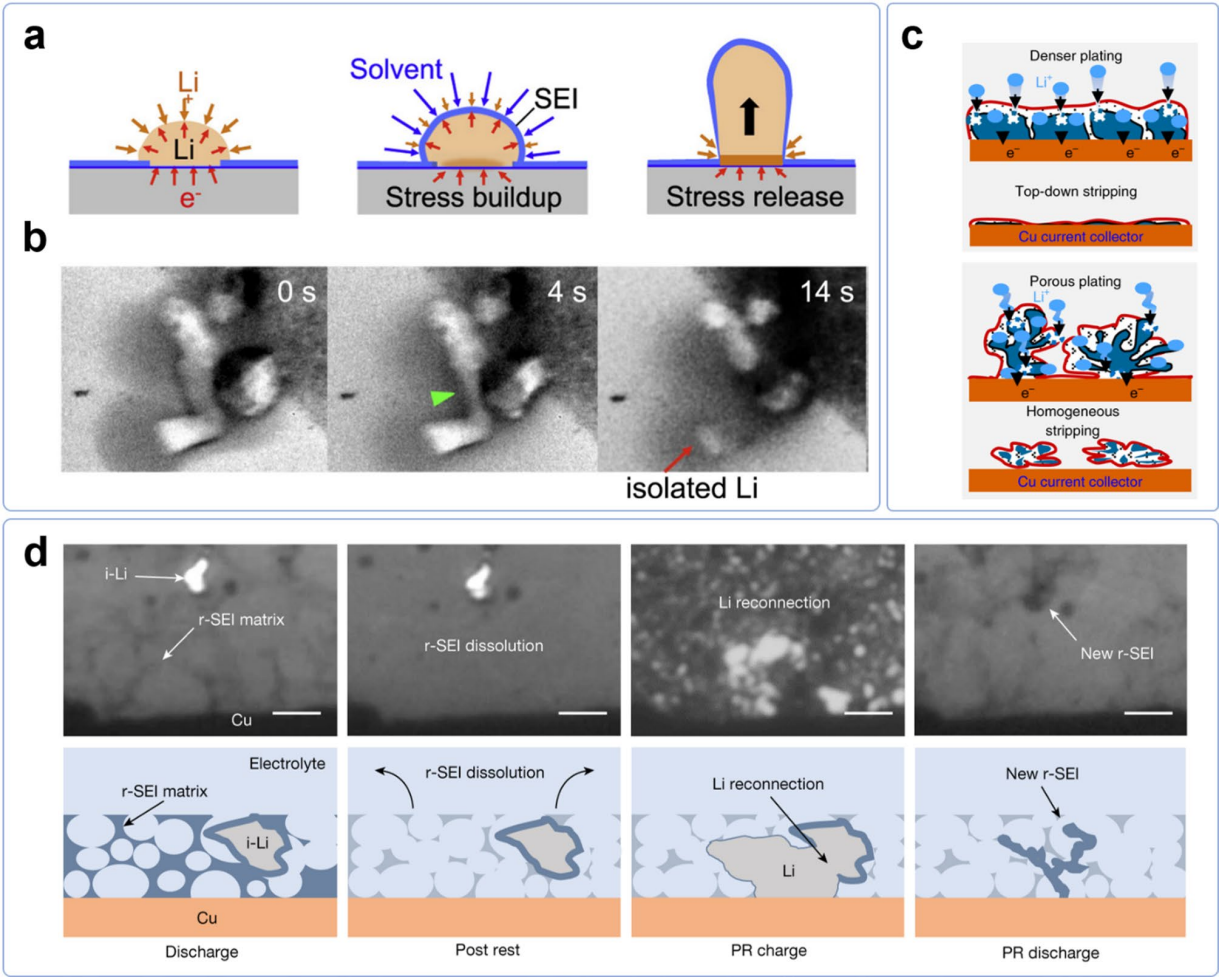
**a**) Schematic diagram illustrating root-growth process. **b**) Evolution of dead Li from root-grown whiskers. Reproduced with permission from Ref. [26]. Copyright 2017, Elsevier. **c**) Schematic diagram illustrating plating/stripping behavior in amide electrolyte (upper panel) and conventional carbonate electrolyte (lower panel), characterized by denser plating/top-down stripping and porous plating/homogeneous stripping, respectively. Reproduced with permission from Ref. [27], CC BY 4.0. **d**) Optical visualization and schematic representation of the suggested mechanism for r-SEI dissolution and i-Li recovery. Reproduced with permission from Ref. [28]. Copyright 2024, Springer Nature

Additionally, a less conspicuous yet equally critical challenge is the formation of dead lithium. This term refers to isolated lithium fragments that become electrically disconnected from the anode during the charge-discharge process. The phenomenon occurs due to incomplete lithium deposition and stripping, resulting in an accumulation of inactive lithium. Over successive cycles, this exacerbates lithium inventory loss and adversely affects both capacity retention and cycling efficiency. Kushiama et al. employed liquid cell transmission electron microscopy (LCTEM) to directly observe the nucleation, growth, and dissolution of lithium metal, providing nanoscale insights into dead lithium formation [26]​. The experimental setup featured a custom-built liquid environmental electrochemical cell (LEEC), designed to maintain a stable liquid electrolyte environment within the TEM system. This apparatus enabled real-time visualization of lithium electrodeposition and stripping, capturing key morphological transitions. Through in-situ LCTEM imaging, the study elucidated that dead lithium predominantly results from the root-growth mechanism of lithium whiskers. The authors, based on their in-situ observations, propose that stress-induced rupture of the SEI layer plays a key role in the evolution of root growth. They suggest that during lithium electrodeposition, the confinement imposed by the SEI leads to stress build-up in the underlying lithium. This stress may eventually cause the SEI to rupture, creating openings through which lithium extrudes from the root, leading to the characteristic root growth mode observed in their experiments (Fig. 3a). As lithium is stripped from root-grown whiskers, dissolution preferentially occurs at the newly formed segments where the SEI is thinner, leading to rapid segmental shrinkage near the base. This frequently causes the whiskers to become electrically disconnected, leaving behind isolated lithium structures (Fig. 3b). Furthermore, the brittle nature of the hollowed-out SEI shell facilitates the detachment of these dead lithium fragments, which are then swept into the electrolyte, forming what the researchers termed “nano-lithium flotsam”. This process underscores the crucial role of SEI evolution and mechanical stress in the irreversible loss of lithium.

More recently, Chen et al. employed a combination of in-situ optical microscopy, transmission electron microscopy (TEM), and titration gas chromatography (TGC) to investigate the formation of dead lithium during the stripping process in lithium metal batteries [29]. Through in-situ optical microscopy, the researchers observed the morphological evolution of lithium deposition and stripping, revealing that inactive lithium accumulates on the anode surface over successive cycles. TEM analysis further confirmed the presence of unreacted metallic lithium encapsulated by the solid-electrolyte interphase, forming electrically isolated dead lithium. Additionally, TGC was utilized to quantify the proportion of dead lithium by measuring hydrogen gas evolution from the reaction between lithium and water. The authors systematically demonstrated that the formation of dead lithium is governed by three key processes: electron transfer within electrodeposited lithium, the conversion of Li⁰ to Li⁺, and lithium ion diffusion through the SEI. Their findings highlight that minimizing dead lithium requires maintaining sufficient electrical contact to ensure continuous electron pathways, optimizing the stripping current density, and developing a uniform, fast ion-conducting SEI to facilitate efficient Li⁺ transport.

A notable study quantitatively evaluated the sources of inactive lithium in a coin cell environment and provided crucial insights into the reversibility of lithium metal anodes [30]. Using titration gas chromatography (TGC), the researchers distinguished between unreacted metallic lithium (Li⁰) and lithium-ion (Li⁺) compounds within the solid-electrolyte interphase. Building on this analysis, the researchers investigated how different electrolyte conditions influence lithium reversibility and the relative contributions of SEI growth and dead lithium formation to capacity loss. Their findings revealed that in electrolytes exhibiting a Coulombic efficiency above 95%, electrolyte decomposition and the subsequent SEI growth were the primary causes of capacity loss, whereas the contribution of dead lithium was relatively minor. As CE increases beyond this threshold, the proportion of inactive lithium attributed to dead lithium becomes significantly smaller, reinforcing the idea that SEI formation, rather than electrically isolated lithium, is the primary driver of irreversibility in high-CE systems. However, it is noteworthy that the deposition/stripping, and degradation behavior of lithium metal anodes can vary significantly depending on cell configuration and operating conditions [31–35]. More specifically, a recent study demonstrated that the amount of dead lithium formation varies depending on the stripping current density [29]. These findings still warrant careful attention to the issue of dead lithium.

Among various strategies to address the issue of dead lithium, one approach is to mitigate its formation at the early stages by optimizing lithium deposition and stripping behavior [27, 36–41]. Wang et al. introduced an amide-based electrolyte, which facilitates the formation of a stable and highly ion-conductive interface [27]. This tailored interfacial chemistry promotes dense and uniform lithium deposition, reducing the porosity of the lithium layer and enhancing electron transport across the electrode. As a result, during the stripping process, lithium is preferentially removed from the uppermost layer rather than being stripped homogeneously throughout the deposit. Such top-down stripping minimizes the risk of isolated lithium regions becoming electrically disconnected, thereby preventing dead lithium formation at its early stage (Fig. 3c). In contrast, in conventional carbonate-based electrolytes, lithium deposition tends to be more porous and uneven, leading to homogeneous stripping throughout the entire lithium layer. This increases the likelihood of lithium islands becoming electrically disconnected, forming inactive dead lithium over repeated cycles. By fundamentally altering the lithium deposition and stripping behavior, the amide-based electrolyte significantly improves cycling stability and achieves high Coulombic efficiency, demonstrating the potential of electrolyte engineering for highly reversible lithium metal batteries.

In a more recent study, Jo et al. demonstrated that uniform lithium deposition can be achieved by structurally modifying the current collector [42]. They introduced a bottom-enriched gradient of Mg seeds into a porous Cu framework via pulse-current electrodeposition, leveraging the strong lithiophilicity of Mg to guide lithium nucleation preferentially at the bottom region. The use of pulse-current electrodeposition enabled precise spatial control over Mg distribution within the 3D host, facilitating the formation of a vertically graded architecture. This architecture effectively guided lithium to plate from the bottom up, mitigating dendritic growth and enhancing electrochemical performance, as evidenced by the extended cycling stability in symmetric cells and the improved lifespan of NCM622-based full cells.

In addition to strategies aimed at preventing dead lithium formation, research has also explored methods to reactivate electrically disconnected lithium, thereby improving overall lithium utilization and extending cycle life [28, 43–46]. Jin et al. proposed a redox-mediated approach for reclaiming inactive lithium using a triiodide/iodide (I₃⁻/I⁻) redox couple. In their study, stannic iodide (SnI₄) was introduced to initiate a redox cycle, where I₃⁻ chemically reacts with inactive lithium, converting it into soluble LiI, which then diffuses to the cathode [43]. Upon oxidation at the cathode, LiI regenerates I₃⁻, enabling continuous lithium recovery over multiple cycles. This reversible redox process significantly mitigates lithium loss and doubles the cycle life of lithium metal batteries employing this strategy.

A recent study by Zhang et al. explored an electrochemical cycling protocol to reactivate isolated lithium (i-Li) by leveraging discharged-state calendar aging​ [28]. By resting lithium-metal batteries in a fully discharged state, they observed an increase in Coulombic efficiency above 100%, indicating capacity recovery. Operando optical microscopy and titration gas chromatography confirmed that lithium previously disconnected from the electrochemical circuit was gradually reincorporated into active cycling (Fig. 3d). The study attributes this recovery to the dissolution of residual SEI shells, which exposes isolated lithium, allowing it to reconnect with the current collector during subsequent charging cycles. This approach facilitates deeper investigation into rest-driven lithium recovery and encourages protocol refinement aimed at improving battery durability.

## Properties of SEI

The solid-electrolyte interphase plays a critical role in stabilizing lithium metal anodes by preventing continuous electrolyte decomposition and influencing lithium deposition behavior. Accordingly, in-depth understanding and optimization of the SEI composition and structure is essential, as these parameters govern its key properties such as mechanical strength, ionic conductivity, and long-term stability. Such understanding informs the rational design of SEI composition and structure, which is central to achieving high reversibility in lithium metal batteries.

### SEI components

#### Organic components

The ideal solid-electrolyte interphase is commonly expected to exhibit strong mechanical properties, high chemical stability, high ionic conductivity, and low electronic conductivity [47, 48]. The components that constitute the SEI play a crucial role in determining these properties [49, 50]. SEI components can be broadly categorized into inorganic and organic species, each possessing distinct physicochemical characteristics (Table 1) [49, 51–61]. Inorganic SEI components generally exhibit superior mechanical properties, such as high shear modulus and Young’s modulus, and greater chemical stability. However, their dense and crystalline nature can hinder lithium-ion transport, making them less favorable for ion conduction [62, 63]. In contrast, organic SEI components typically show inferior mechanical and chemical stability, but their amorphous or porous structure can facilitate relatively easier lithium-ion transport. Nonetheless, their practical role in stabilizing lithium metal anodes remains questionable. Recent studies have reported a negative correlation between the presence of organic components in the SEI and the reversibility of the lithium metal anode [64, 65], which suggests that organic components cannot be key constituents in ensuring the full reversibility of lithium metal.

**Table 1:** Physical properties and Li^+^ transport characteristics of SEI components

	Shear Modulus (GPa)	Young’s Modulus (GPa)	Bandgap (eV)	Ionic Conductivity (S cm^− 1^)	Li^+^ Migration Energy Barrier (eV)
Organics (ROCO_2_Li)	-	< 1 [52]	-	< 10^− 9^ [52]	0.76 [52]
LiF	55.1 [51]	65.0 [52]	8.9 [54]	10^− 27^ [57]	0.56 [57]
Li_2_O	45.6 [51]	169.0 [52]	4.7 [54]	10^− 12^ [58]	0.70 [58]
Li_2_CO_3_	28.9 [51]	75.0 [52]	4.7 [54]	10^− 11^–10^− 8^ [52]	0.235–0.683 [61]
Li_2_S	-	82.6 [52]	3.66 [55]	< 10^− 10^ [59]	0.57 [59]
Li_3_N	-	48 [53]	1.1 [56]	10^− 4^ [60]	0.007–0.038 [60]

**Fig. 4:**
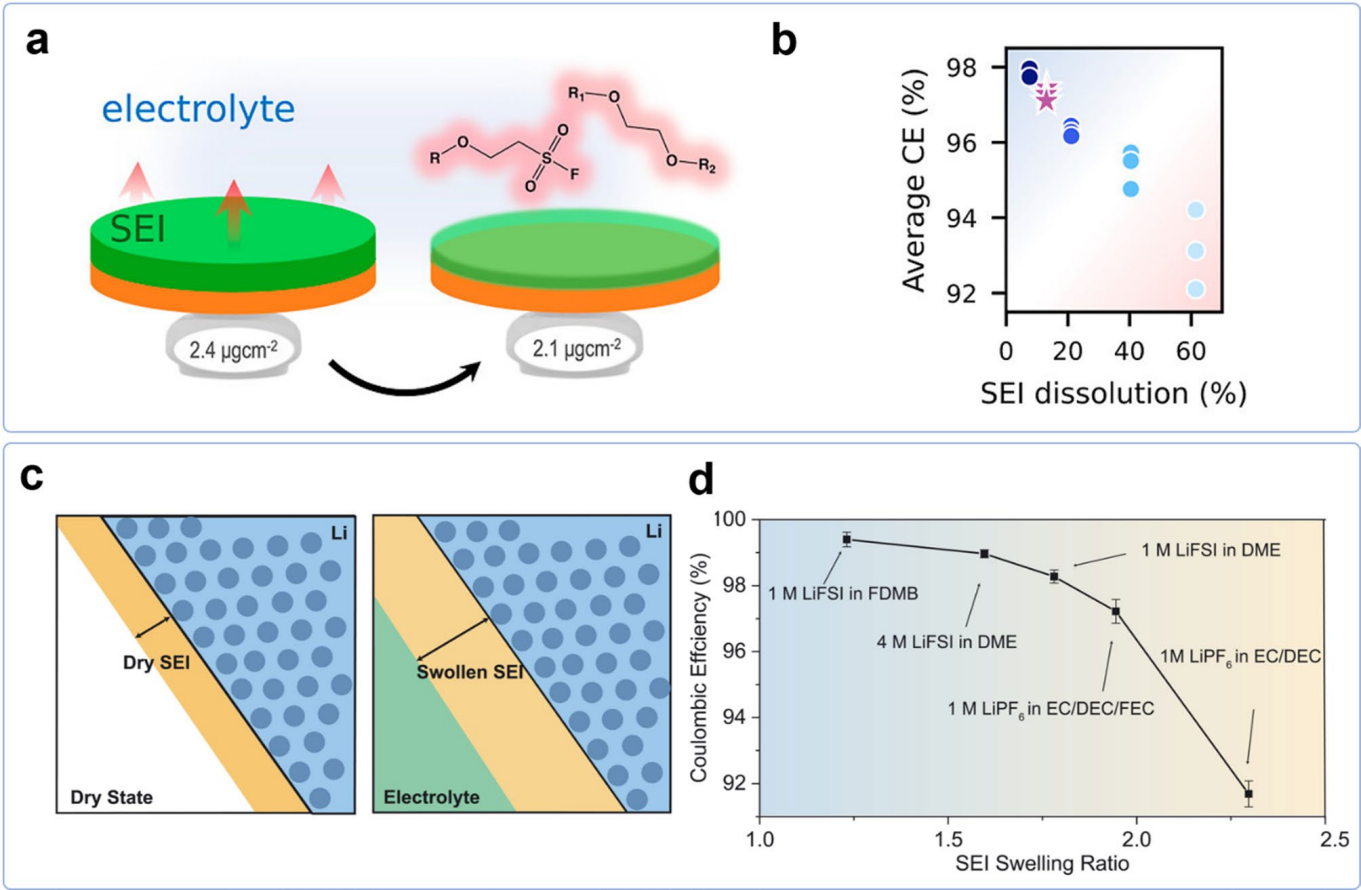
**a**) Quantification of SEI dissolution using EQCM. **b**) Negative correlation between SEI dissolution and average CE. Reprinted with permission from Ref. 64. Copyright 2023, American Chemical Society. **c**) Schematic diagram illustrating swelling of SEI. **d**) Negative correlation between SEI swelling ratio and CE. Reproduced with permission from Ref. 65. Copyright 2022, The American Association for the Advancement of Science

Sayavong et al. investigated the dissolution behavior of the solid-electrolyte interphase (SEI) in lithium metal batteries, employing electrochemical quartz crystal microbalance (EQCM) to systematically quantify SEI mass loss (Fig. 4a) [64]. Their study revealed that organic components within the SEI exhibit notable solubility in the electrolyte, leading to continuous SEI dissolution and reformation. This process results in excessive lithium consumption and increased SEI thickness over cycles, ultimately reducing the CE and degrading battery performance (Fig. 4b). The research demonstrates that SEIs rich in lithium fluoride (LiF) exhibit lower solubility and better passivation, effectively reducing unwanted side reactions. This underscores the importance of designing SEIs with a higher fraction of inorganic, stable components to enhance the long-term cyclability of lithium metal batteries.

Studies reporting SEI swelling have also shown a negative correlation between organic components and Coulombic efficiency [65]. Zhang et al. investigated the swelling behavior of the solid-electrolyte interphase in lithium metal batteries (Fig. 4c). They found that organic components within the SEI contribute significantly to swelling, which in turn compromises battery performance. Their study revealed that organic-rich SEIs absorb more electrolyte, leading to increased thickness and reduced mechanical stability, ultimately exposing more lithium to parasitic reactions. This swelling effect correlates strongly with lower CE, as batteries with highly swollen SEIs exhibit faster degradation and higher lithium consumption (Fig. 4d). Conversely, SEIs with more inorganic content, such as LiF and Li_2_O, show reduced swelling and better passivation, leading to improved cycling stability. These findings emphasize that minimizing organic components in the SEI while incorporating more inorganic species (e.g., LiF, Li_2_O, Li_2_CO_3_) is crucial for preventing excessive swelling, achieving high CE, and enhancing lithium metal battery performance.

#### Inorganic components: focus on LiF and Li_2_O

Inorganic components, which are more stable in organic electrolytes and provide superior passivation, are considered to play a crucial role in ensuring the reversibility of LMBs. The inorganic species in the SEI include a diverse range of compounds, such as LiF, Li₂CO₃, Li₂O, Li₃N. In general, each component in the solid-electrolyte interphase is not present as a pure substance, making it challenging to validate the effects of individual constituents [66, 67]. Despite these challenges, a growing body of research has reported that LiF-rich SEI consistently contributes to improved electrochemical performance, reinforcing the notion that LiF is increasingly regarded as a key component for ensuring the reversibility of lithium metal anodes [68–71].

LiF is considered advantageous as a solid-electrolyte interphase component due to its high mechanical strength, as represented by its shear modulus and Young’s modulus, as well as its chemical inertness, low electronic conductivity, and high interfacial energy. However, LiF also has low bulk ionic conductivity, which raises concerns about its suitability as an SEI component, given the importance of efficient charge transport in electrochemical cells. Nevertheless, its impact on ion transport within the SEI is not solely determined by its bulk properties. In polycrystalline structures, grain boundaries can serve as preferential pathways for ion transport, facilitating relatively faster Li-ion conduction compared to the bulk material [72, 73]. In a related note, Zhang et al. reported a synergetic effect of LiF and Li_2_CO_3_ interfaces caused by a space charge accumulation and higher ionic carrier concentration which not only facilitates Li-ion migration across boundaries but also prevents undesired electrolyte decomposition [74]. Computational studies, including first-principles calculations, have also supported faster ion transport through grain boundaries, demonstrating lithium diffusion in polycrystalline SEI components [75].

Given this context, LiF exhibits a combination of beneficial physicochemical properties and potential for moderate ionic transport via grain boundary, making it an ideal SEI component. Numerous studies have reported that the implementation of LiF-rich SEI on lithium metal anodes leads to improved reversibility [68]. This enhancement is primarily linked to the ability of LiF-rich SEI to regulate lithium deposition morphology, promoting uniform plating [76], while also providing effective passivation that stabilizes the electrode interface [77, 78].

**Fig. 5:**
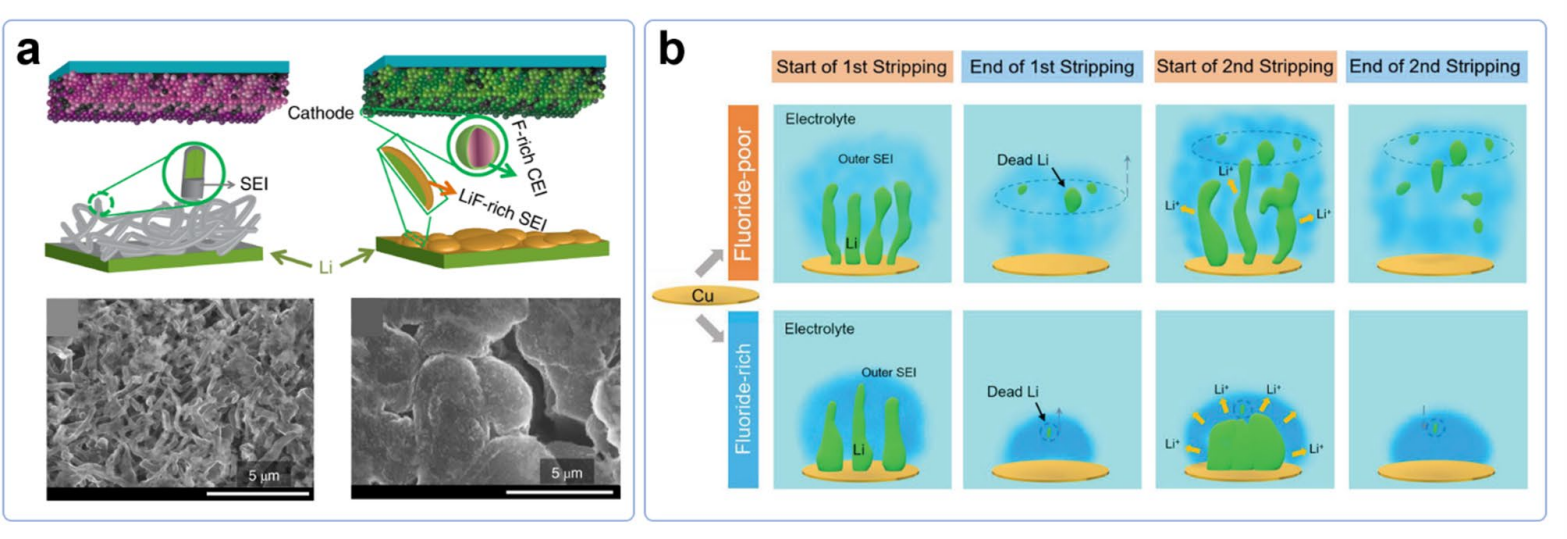
**a**) Schematic representation and optical image showing dendritic and levelled Li deposition in conventional carbonate electrolyte and all-fluorinated electrolyte, respectively. Reproduced with permission from Ref. [76]. Copyright 2018, Springer Nature. **b**) Schematic representation illustrating different plating and stripping behavior under Fluorine-poor and Fluorine-rich SEI. Reproduced with permission from Ref. [79], CC BY

Specifically, suppressing the dendritic growth of lithium and ensuring a flat deposition have been recognized as one of the most critical factors in achieving high reversibility of lithium metal since the early stages of research. Given this context, there have been efforts to rationally explain how a LiF-rich SEI contributes to smooth lithium deposition. Fan et al. introduced a LiF-rich SEI using an all-fluorinated electrolyte, which resulted in dense and smooth lithium deposition (Fig. 5a) [76]. The authors attributed this effect to the high interfacial energy of LiF, which facilitates Li-ion migration along the interface. This energetic preference encourages parallel lithium deposition, rather than vertical dendritic growth, leading to a more uniform lithium morphology.

Gong et al. further investigated the role of a LiF-rich SEI in achieving smooth lithium deposition using operando liquid-cell transmission electron microscopy (LC-STEM) [79]. Their real-time imaging provided direct evidence that a fluoride-rich SEI leads to a denser and more uniform lithium plating morphology, which is more conducive to leveled lithium deposition. By comparing lithium cycling behavior across fluoride-poor and fluoride-rich interphases, they observed that the latter significantly suppresses the formation of dead lithium and enhances lithium stripping uniformity (Fig. 5b). Supporting these imaging results, secondary ion mass spectrometry (SIMS) and nuclear magnetic resonance (NMR) spectroscopy confirmed an enrichment of lithium fluoride in the SEI, which contributes to a more homogeneous ionic conductivity distribution. It is explained this uniform conductivity prevents localized lithium accumulation and promotes a smoother, more stable plating/stripping process.

Among the inorganic constituents of the solid-electrolyte interphase, Li₂O constitutes a major fraction alongside LiF. Gallant’s research group highlighted that although Li₂O is a major component of the inner-layer SEI, its transport characteristics in realistic battery environments remain poorly understood [80]. To address this, they systematically constructed single-component SEIs composed of Li₂O and LiF by exposing lithium metal to controlled gas-phase reactions using O_2_ for Li_2_O and NF_3_ for LiF. These artificially synthesized SEIs were then analyzed to extract their ionic conductivity, charge carrier concentration, and diffusivity using electrochemical impedance spectroscopy (EIS). Their findings demonstrated the superior transport properties of Li₂O, showing that its ionic conductivity and charge carrier diffusivity were higher than those of LiF. This suggests that Li₂O-enriched SEIs may offer improved lithium transport and stability, providing new insights for the rational design of SEI structures in lithium metal batteries.

**Fig. 6:**
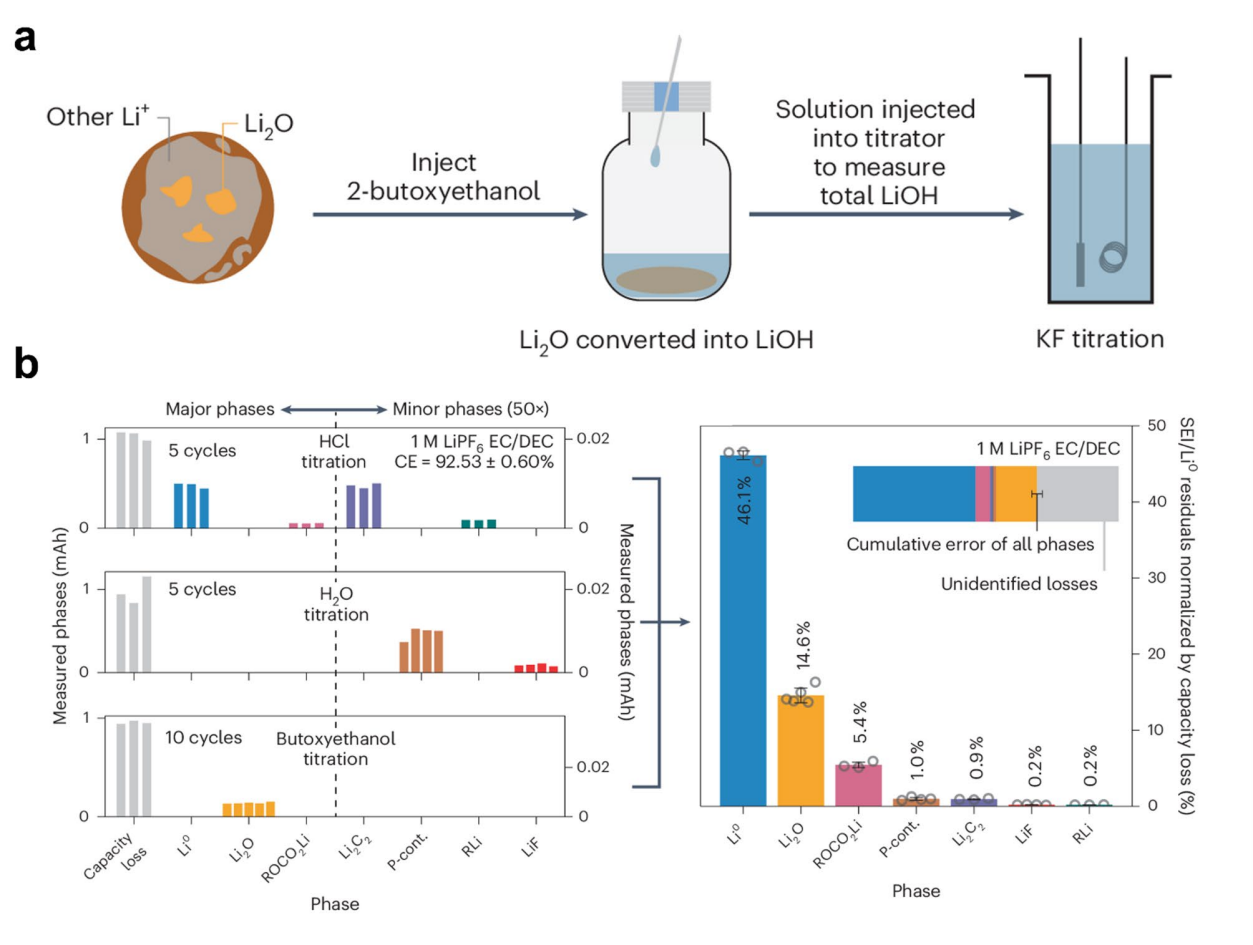
**a**) Illustration of the experimental process for quantifying Li_2_O. **b**) Quantification of inactive lithium and SEI-constituting phase including Li_2_O. Reproduced with permission from Ref. [81]. Copyright 2024, Springer Nature

Building upon this, the same research group sought to further investigate the relative significance of Li_2_O and LiF in SEI composition and its impact on lithium metal battery performance [81]. Given that Li_2_O is the second major ionic phase in SEI models and has been associated with improved lithium-ion transport, they aimed to accurately quantify its presence in cycled Li anodes—an area previously hindered by the lack of a selective analytical technique. To systematically construct SEIs with varying Li₂O contents, they leveraged a broad range of electrolyte compositions that naturally induce different SEI chemistries, spanning fluorinated, oxygenated, and hybrid systems. By tuning solvent and salt selection, they were able to generate SEIs with diverse Li₂O-to-LiF ratios, enabling a controlled comparison of how these phases influence Coulombic efficiency. To accurately quantify these SEI components, they developed an alcohol-based titration method followed by Karl Fischer analysis, allowing for the selective detection of Li₂O alongside other key SEI phases, including LiF, Li₃N, sulfur- and boron-containing phases, Li₂CO₃, and inactive lithium (Li⁰) (Fig. 6a and b). This methodology, applied across ten diverse electrolytes, revealed that Li₂O, rather than LiF, is the most consistently abundant SEI phase at high CE. These findings challenge the conventional assumption that LiF enrichment was the sole key factor in achieving high CE. Instead, they demonstrate that oxygenation of the SEI, facilitated by oxygenated solvents and salts, can yield CE values exceeding 99%, rivaling fluorinated electrolyte systems. This study not only provides a more comprehensive framework for SEI phase quantification but also highlights SEI oxygenation as a promising yet underexplored pathway for rational electrolyte design and lithium metal battery stabilization.

### SEI structure

Regarding the structure of the SEI, the mosaic model proposed by Peled et al. has been widely accepted [82]. This model depicts the SEI as a heterogeneous assembly of inorganic components such as Li₂O, LiF, and Li₂CO₃, along with organic polymeric species, distributed in a mosaic-like pattern. Recent advancements in cryogenic transmission electron microscopy (cryo-TEM) have enabled direct observation of the SEI in lithium metal batteries, providing unprecedented insights into its complex nanostructure [83]. By rapidly freezing samples to cryogenic temperatures, cryo-TEM preserves the native state of beam-sensitive materials like the SEI, allowing for high-resolution imaging without inducing damage. With such advancement of analytical tools, the structural analysis of the solid-electrolyte interphase has become increasingly sophisticated, leading to the discovery of new SEI architectures. In a recent perspective, Ben Jagger et al. classified SEI nanostructures into five distinct models based on emerging research: the mosaic model, refined mosaic model, amorphous model, multilayered model, and extended SEI model [84]. These newly proposed models exhibit significant structural distinctions from the conventional mosaic model. Notably, the amorphous model highlights the presence of a disordered SEI layer, while the extended SEI model reveals a thick, porous structure extending up to 500 nm. These findings suggest that SEI formation is highly dependent on electrolyte conditions, leading to a diverse range of SEI architectures across different electrochemical environments. Such insights underscore the complexity of SEI evolution.

**Fig. 7:**
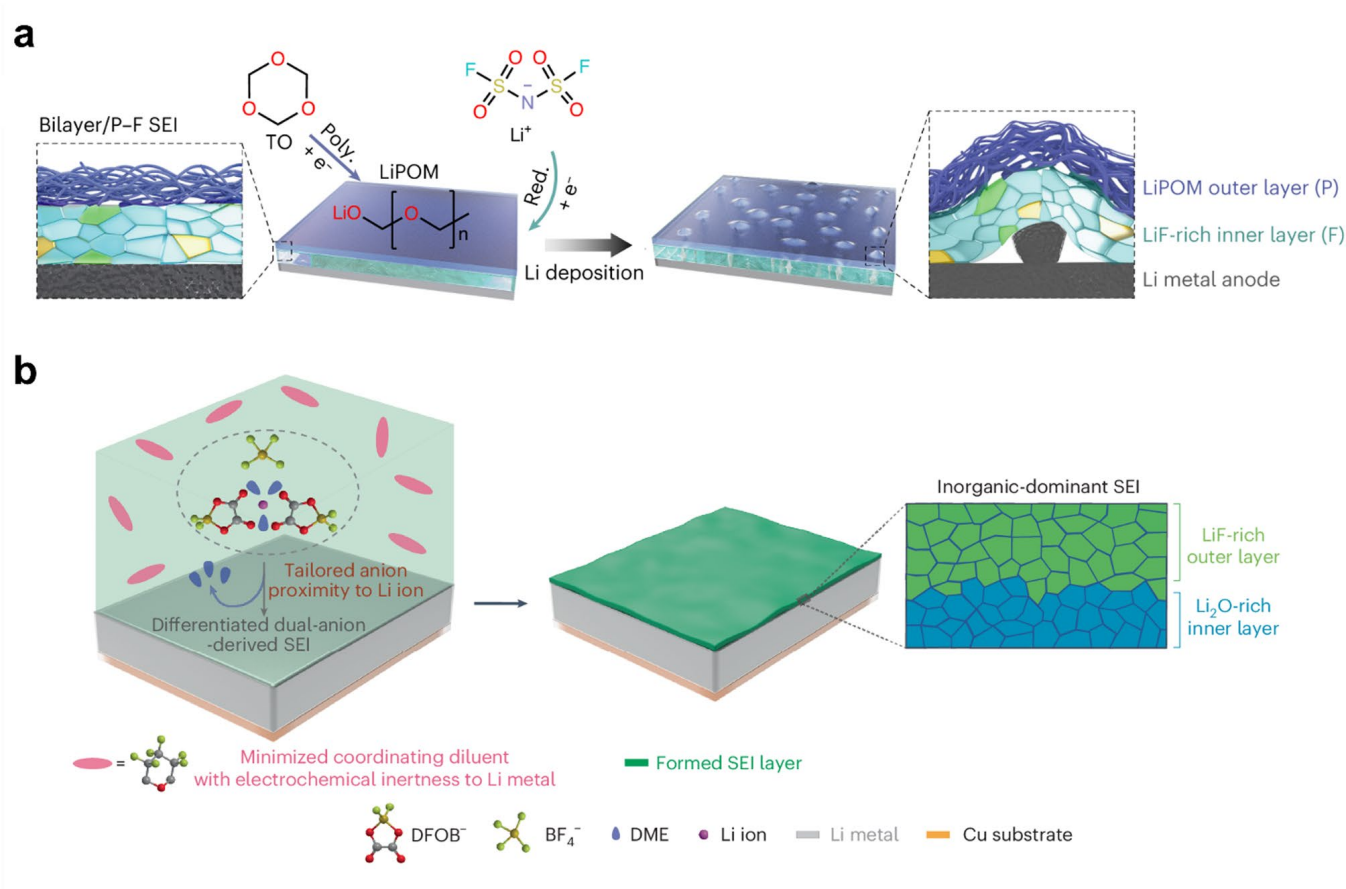
**a**) Schematic illustration of the formation of bilayer SEI composed of LiF and LiPOM under trioxane-modified electrolyte. Reproduced with permission from Ref. [86]. Copyright 2023, Springer Nature. **b**) Schematic illustration of the formation of inorganic dominant SEI composed of Li_2_O-rich inner layer and LiF-rich outer layer. Reproduced with permission from Ref. [87]. Copyright 2024, Springer Nature

Recent studies have focused not only on the overall structural characteristics of the solid-electrolyte interphase but also on the unique structural relationships among its various components. Zhang et al. investigated this aspect by constructing a bilayer SEI with distinct internal and external compositions [85]. Using isosorbide dinitrate (ISDN) as an additive in a localized high-concentration electrolyte, they demonstrated that LiN_x_O_y_ preferentially formed in the top layer, while LiF dominated the bottom layer near the lithium metal anode. This layered architecture provided a more uniform lithium-ion transport pathway, reducing uneven lithium deposition and mitigating continuous SEI reconstruction. As a result, the bilayer SEI significantly extended the cycle life of lithium metal batteries, achieving a threefold improvement over conventional anion-derived SEI systems. In a subsequent study, Zhang et al. reported a bilayer SEI structure using a trioxane (TO)-modulated electrolyte, where the inner layer was LiF-rich, promoting homogeneous Li-ion transport, while the outer layer contained Li polyoxymethylene (LiPOM) to enhance mechanical stability (Fig. 7a) [86]. This strategic design mitigated SEI cracking and reconstruction, reducing side reactions and improving lithium plating/stripping reversibility.

Li et al. introduced a distinctive approach to SEI engineering by developing a hierarchically structured inorganic-dominant SEI [87]. By employing a minimized coordinating fluorinated cyclic ether diluent (HFTHP) in a dual-salt electrolyte, they precisely regulated the solvation environment of Li ions, leading to a bilayer SEI with differentiated inorganic compositions. The inner SEI layer was Li₂O-rich, providing superior ionic conductivity for uniform lithium deposition, while the outer layer was LiF-rich, offering enhanced mechanical stability and passivation against electrolyte decomposition​ (Fig. 7b). This tailored SEI structure significantly suppressed parasitic reactions, reducing self-discharge and improving long-term battery stability.

According to the research by Gallant’s research group, which demonstrated the high potential of Li₂O through a statistical approach, not only the composition of the SEI but also its structural characteristics, such as the uniform spatial distribution of its components, were found to play a crucial role in enhancing its functionality [81]. Given this context, studies that focus on precisely controlling the spatial arrangement of SEI components represent a promising avenue for further enhancing SEI performance, ultimately contributing to the advancement of high-performance lithium metal batteries.

## Strategies for advanced SEI engineering

### Modifying solvation structure

Solvation structure engineering offers a powerful means of improving the electrochemical performance and stability of lithium metal batteries. The way lithium ions interact with surrounding solvent molecules and anions fundamentally dictates electrolyte properties, influencing lithium-ion transport, interfacial reactions, and the formation of the solid-electrolyte interphase [88]. Uncontrolled solvation structures often lead to excessive electrolyte decomposition, sluggish ion transport, and unstable electrode interfaces, ultimately limiting battery efficiency and cycle life. Addressing these challenges requires a precise understanding of solvation environments and their impact on electrolyte behavior.

**Fig. 8:**
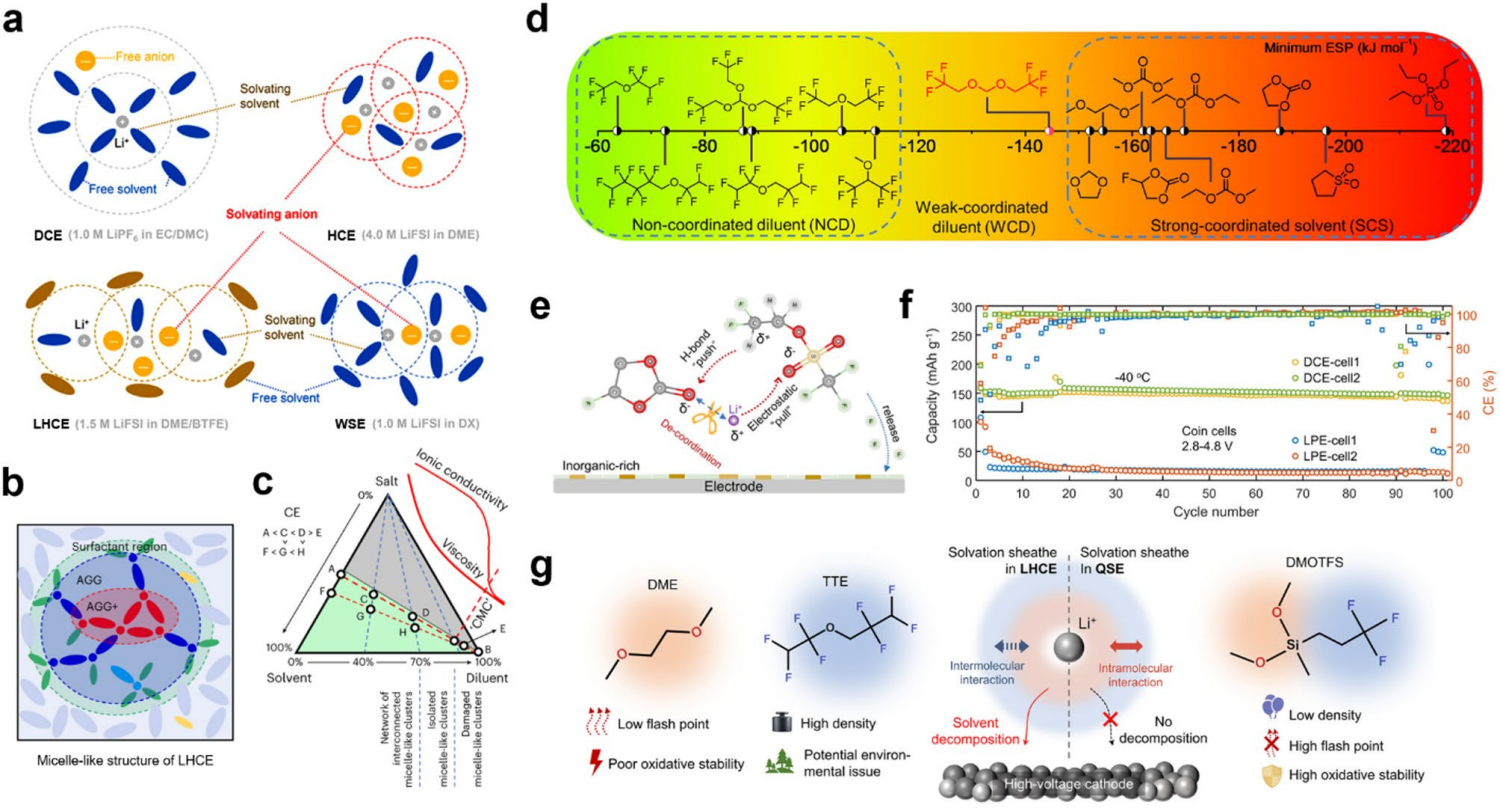
**a**) Schematic illustration of the solvation structures of a dilute conventional electrolyte (DCE), HCE, LHCE, and WSE. Reproduced with permission from Ref. [89]. Copyright 2021, John Wiley & Sons, Inc. **b**) Schematic illustration of the micelle-like structure of LHCE. **c**) LHCE design criteria based on the ternary phase diagram of salt, solvent, and diluent. Reproduced with permission from Ref. [90]. Copyright 2023, Springer Nature. **d**) Calculated minimum electrostatic potential (ESP) values of reported diluents and solvents. Reproduced with permission from Ref. [91]. Copyright 2023, Elsevier. **e**) Schematic illustration of the “push–pull” mechanism of the DTF cosolvent. **f**) Cycling performance of a Li||NMC811 full-cell with the electrolyte at − 40 °C under a charge/discharge rate of 0.067/0.2 C. Reproduced with permission from Ref. [92]. Copyright 2024, American Chemical Society. **g**) Comparison between the semisolvated sole-solvent electrolyte (QSE) and LHCE. Reproduced with permission from Ref. [93]. Copyright 2023, American Chemical Society

To address these challenges, electrolyte development has evolved from conventional solvent-rich systems toward more regulated solvation environments, including high-concentration electrolytes (HCEs), localized high-concentration electrolytes (LHCEs), and weakly solvating electrolytes (WSEs). This progression in solvation structure reflects a continuous shift from solvent-dominated coordination environments to those increasingly governed by anion-rich interactions. The conceptual framework of this evolution is illustrated in Fig. 8a, which summarizes how each electrolyte type restructures the local coordination of lithium ions [89]. These distinct strategies offer alternative pathways to optimize Li⁺–solvent and Li⁺–anion interactions, ultimately aiming to improve interfacial stability and overall electrochemical performance.

#### Optimizing solvation structure in LHCEs: micelle-like clusters, diluent tuning, and stability control

Localized high-concentration electrolytes have emerged as a key strategy for stabilizing lithium metal anodes by leveraging solvation structure engineering to address the limitations of conventional electrolytes [94–97]. Their development stems from high-concentration electrolytes (HCEs), which reduce solvent reactivity by promoting anion-rich coordination environments. In HCEs, lithium ions are primarily coordinated within contact ion pairs (CIPs) and aggregates (AGGs), suppressing solvent-induced side reactions and enabling the formation of a highly inorganic SEI [98–101]. However, the excessive viscosity and limited ionic conductivity of HCEs restrict their practical implementation [94, 100].

LHCEs were designed to retain the benefits of HCEs while restoring electrolyte fluidity through the introduction of non-coordinating diluents [94]. These diluents enable localized high salt concentrations while preventing the bulk electrolyte from becoming overly viscous. Recent studies indicate that, rather than forming a random dispersion of solvated species, LHCEs adopt a micelle-like solvation structure (Fig. 8b) [90]. In this arrangement, solvents act as surfactants, stabilizing highly concentrated ion aggregates at the core, whereas the diluent phase remains largely excluded from the primary solvation sheath. This self-assembled solvation architecture induces a salt concentration gradient, with highly coordinated ion-pair species dominating the core, promoting the formation of a dense, inorganic-rich SEI. However, the stability of this micelle-like structure is highly sensitive to electrolyte composition (Fig. 8c). Excessive diluent disrupts these clusters, leading to phase separation, while insufficient diluent increases viscosity, hindering lithium-ion mobility. To preserve these microstructures while maintaining ionic conductivity, an optimal diluent content range of 40–70 mol% has been identified as crucial. Beyond simply controlling diluent ratios, electrolyte formulation must also maximize local salt concentration, aligning with the solubility line in the ternary phase diagram to reinforce the presence of highly coordinated lithium-ion clusters.

Further refinement of LHCEs requires a deeper understanding of diluent–anion interactions, which play a crucial role in maintaining electrolyte homogeneity. Wu et al. demonstrated that the maximum electrostatic potential energy (ESP_max_) of a diluent serve as a key criterion for forming a single-phase LHCE, as it reflects the interaction strength between the diluent and the electronegative FSI^–^ anion [102]. Experimental findings show that diluents with ESP_max_ below 25 kcal mol⁻¹ fail to stabilize LHCEs in the LiFSI-DME system due to insufficient interaction with FSI^–^, leading to phase separation. However, an excessively strong interaction is also problematic, as it disrupts Li⁺–DME–FSI^–^ coordination network, increasing the presence of free DME and accelerating electrolyte decomposition. Thus, selecting an appropriate diluent is critical not only for phase stability but also for preserving the structural integrity of solvation clusters. Among various candidates, 2 H,3 H-decafluoropentane (HFC) has demonstrated its ability to suppress solvent de-coordination while stabilizing Li⁺–DME–FSI^–^ structure, ultimately improving lithium metal reversibility and oxidation resistance in high-voltage systems.

Beyond maintaining solvation structure integrity, tuning diluent properties plays a crucial role in lithium-ion desolvation kinetics. Zhao et al. introduced weakly coordinating diluents, which exist in an intermediate electrostatic potential range between non-coordinating diluents (ESP_min_ > − 120 kJ mol^–1^) and strongly coordinating solvents (ESP_min_ < − 150 kJ mol^–1^) (Fig. 8d) [91]. Unlike non-coordinating diluents, which remain largely inert in solvation structures, weakly coordinating diluents partially participate in the solvation sheath, subtly weakening Li⁺–anion interactions without disrupting ionic aggregation, though they cannot dissolve salts on their own. This approach has been shown to lower desolvation energy, accelerate lithium-ion transport, and enhance anion decomposition kinetics, ultimately leading to the formation of a more stable, highly inorganic Li₂O-rich SEI. Zhao et al. demonstrated that this solvation tuning strategy increased Coulombic efficiency to 99.72%, highlighting the importance of precise control over solvation interactions in LHCE design. Cui et al. developed a push-pull electrolyte to optimize the desolvation barrier while maintaining an anion-rich solvation structure (Fig. 8e) [92]. ESP screening identified 2,2-difluoroethyl trifluoromethanesulfonate (DTF) as an effective push-pull mediator. The sulfonyl moiety, with a moderate ESP_min_, provides weak Li^+^ solvation ability, allowing Li^+^ to be competitively pulled from solvent coordination without disrupting the anion-rich solvation. At the same time, the difluoromethyl group, characterized by a high ESP_max_, repels solvent molecules from Li^+^ during desolvation through competitive hydrogen bonding. This synergistic mechanism modulates the solvation environment and enhances charge transfer kinetics, enabling stable operation at low temperatures (Fig. 8f).

Another important aspect of solvation structure engineering is preventing solvent de-coordination, which can negatively impact electrolyte longevity [102, 103]. While coordinated solvents within the lithium solvation sheath benefit from a reduced highest occupied molecular orbital (HOMO) level, making them less prone to oxidation [104, 105], de-coordinated solvents become highly reactive, leading to degradation over time. To further stabilize solvation dynamics, semisolvated sole-solvent electrolytes (QSEs) have been explored as a promising complementary strategy. Unlike conventional LHCEs, which rely on separate solvents and cosolvents, QSEs integrate solvating and nonsolvating segments within a single molecule, offering intrinsic stability by preventing solvent dissociation (Fig. 8g). This approach reinforces solvation dynamics and limits excessive solvent de-coordination, providing a new pathway for enhancing electrolyte longevity while maintaining anion-rich solvation structures [93].

A final consideration in solvation structure engineering is the environmental impact of the electrolyte components. Most of the LHCE formulations employ highly fluorinated nonsolvating diluents due to their strong electron-withdrawing effect which enables a non-coordinating character and high electrochemical stability [97, 102, 106–109]. However, the environmental persistence and potential bioaccumulation of these substances have raised growing concerns [110]. Accordingly, fluorine-free diluents that preserve effective solvation structures are increasingly regarded as viable and environmentally responsible alternatives [107, 111, 112].

#### Solvation control in weakly solvating electrolytes

In LHCEs, diluent molecules lack sufficient coordination strength to dissolve Li salts, necessitating the incorporation of highly solvating yet often unstable solvents [113, 114]. This intrinsic limitation has driven the development of weakly solvating electrolytes (WSEs), where Li^+^–solvent interactions are deliberately weakened to promote anion-rich solvation structures, even in single-salt-single-solvent systems. Such solvation environments facilitate the formation of anion-derived, inorganic-rich SEI layers, which mitigate lithium corrosion and enhance CE, while simultaneously suppress Al current collector corrosion by reducing the reactivity of free anions [113]. However, implementing WSEs in practice presents several challenges. Excessively weak Li^+^ coordination can lead to large ion aggregates, which disrupt ionic transport and increase overpotential. Moreover, unlike in HCEs and LHCEs, where oxidation stability is enhanced through strong Li^+^–solvent coordination, WSEs leave a significant fraction of free solvent molecules uncoordinated, making them vulnerable to oxidative degradation under high-voltage conditions. These trade-offs necessitate a delicate equilibrium in solvation strength, driving efforts to refine solvent properties through molecular engineering and optimize solvation structures for improved performance.

**Fig. 9:**
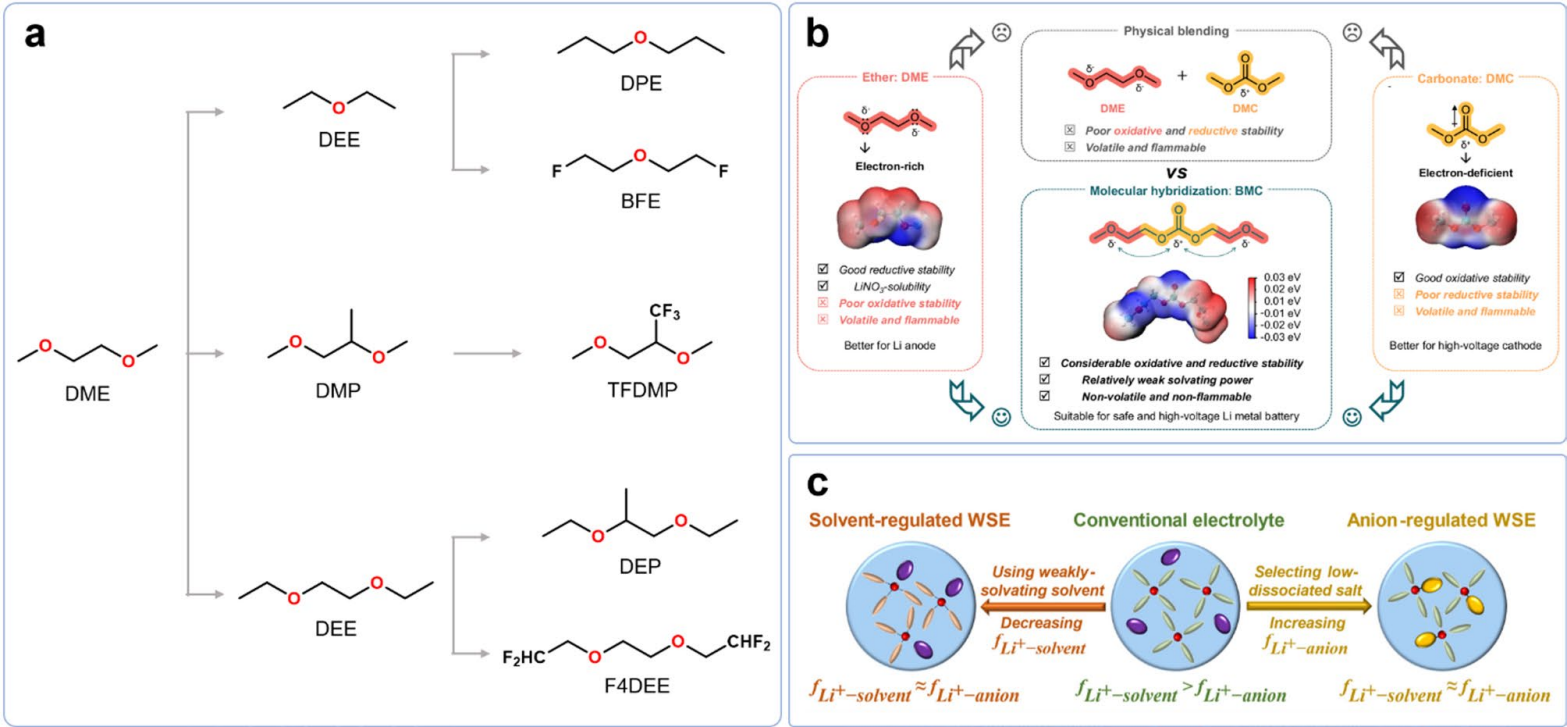
**a**) Molecular engineering of weakly solvent molecules. **b**) Comparison of physical blending and molecular hybridization approaches. Reproduced with permission from Ref. [121], CC BY 4.0. **c**) Solvation structure comparison between two types of WSEs: solvent-regulated and anion-derived WSE. Reproduced with permission from Ref. [122]. Copyright 2022, John Wiley & Sons, Inc

Structural modifications of solvent molecules have been explored to fine-tune solvation power while simultaneously improve oxidative stability (Fig. 9a) [114–117]. A key aspect of this approach is the interplay of steric and electronic effects, which can be leveraged through both functional group substitutions and selective α-H atom modifications. Since α-H atoms in ether molecules are highly susceptible to nucleophilic attack, resulting in poor chemical oxidation stability, their targeted substitution enhances molecular stability while simultaneously influencing solvation properties. Park et al. introduced 1,2-dimethoxypropane (DMP) as a sterically hindered weakly solvating solvent [118]. Compared to 1,2-dimethoxyethane (DME), DMP introduces a methyl (-CH_3_) group at an inner α-H atom, which induces steric hindrance and reduces the accessibility of ethereal oxygen atoms for Li^+^ coordination. This steric constraint weakens solvation power, promoting the formation of an anion-driven SEI layer and facilitating highly reversible Li plating/stripping behavior. Further substituting the methyl group with a trifluoromethyl (-CF_3_) group to form 1,1,1-trifluoro-2,3-dimethoxypropane (TFDMP) highlights the interplay of steric and electronic effects [119]. While the methyl group acts as a weak electron donor that increases the overall electron density of the system, the strongly electron-withdrawing trifluoromethyl group enhances oxidation stability by lowering the HOMO energy level and promotes CIP and AGG formation by reducing the electron density around the coordinating oxygen. Although the strong Li⁺–FSI⁻ interactions slightly reduce ionic conductivity, the electrolyte still maintains a relatively high value of 7.4 mS cm⁻¹, surpassing other fluorinated ether-based electrolytes [113, 114]. Furthermore, progressive methylation of DME was explored, and among the resulting structures, 1,2-diethoxypropane (DEP) with selective methylation at one inner and two outer α-H atoms exhibited the best balance of oxidation stability and ionic conductivity while supporting Li plating/stripping CE values of > 99.7% [120].

Fluorination has been widely employed to modulate Li^+^–solvent interactions; however, precise control over the degree of fluorination is essential to balance solvation strength and ionic transport properties. Yu et al. introduced 1,2-diethoxyethane (DEE) as a weakly coordinating backbone, incorporating β-fluorination to regulate solvation strength while retaining ether-based Li^+^ coordination ability [123]. A systematic study of fluorination revealed that partially fluorinated difluoro (-CHF_2_) groups impart local polarity that moderates solvation, whereas fully fluorinated trifluoro (-CF_3_) groups excessively weaken coordination, leading to conductivity loss. The optimized F4DEE and F5DEE electrolytes achieved CE values of ~ 99.5%, highlighting the effectiveness of partial fluorination in controlling solvation power. Similarly, Zhang et al. demonstrated that monofluoride (-CH_2_F) substitution further promotes fast ion transport, particularly at low temperatures (–30 ℃) [124]. Unlike difluoro and trifluoro groups, which strongly withdraw electron density and weaken Li^+^ coordination, monofluoride groups strike a balance, maintaining localized electron density near coordinating oxygen atom. Moreover, monofluoride groups occupy more localized electrons compared to difluoro and trifluoro counterparts. This structural feature benefiting from strong Li–O and Li–F enables the formation of stable five-membered ring coordination structures around Li^+^. Therefore, the monofluoride bis(2-fluoroethyl) ether (BFE) electrolytes exhibited enhanced ionic conductivity and improved fast-charging performance, underscoring the importance of precisely adjusting fluorination levels to optimize both transport and interfacial properties.

Hybrid solvent designs introduce a new dimension to solvation engineering, offering properties unattainable through simple solvent blending. Chen et al. incorporated ether and carbonate functionalities within a single molecular framework, harnessing the complementary properties of both solvent classes to enhance oxidative and reductive stability (Fig. 9b) [121]. Bis(2-methoxyethyl) carbonate (BMC), which integrates electron-donating ether segments with electron-withdrawing carbonate moieties, redistributes charge density and alters the electrostatic potential distribution. The ESP_min_ shift toward the carbonyl oxygen reduces Li^+^ coordination affinity compared to conventional ether (DME) or carbonate (DMC) solvents. The weaker coordination, coupled with steric hindrance from the larger molecular structure of BMC, limits excessive Li^+^ coordination, thereby favoring anion-dominated solvation structures while ensuring stability on both electrodes.

While solvent modification has been a primary approach, Jiang et al. proposed a new WSE system where anion selection dictates solvation behavior [122]. By employing salts with high dissociation energy, an anion-rich solvation shell was constructed, leading to the preferential formation of anion-derived SEI and CEI (Fig. 9c). Among various salts (LiFSI, LiTFSI, LiDFOB, LiPF_6_, LiBF_4_), Li^+^–BF_4_^–^ interactions exhibited the strongest electrostatic attraction, effectively shifting the solvation equilibrium toward anion-dominated coordination. These findings suggest that beyond solvent design, salt selection plays a pivotal role in modulating solvation behavior and interfacial stability.

In WSEs, oxidative degradation of uncoordinated solvent molecules remains a major challenge, necessitating solvation structures that not only support SEI stability but also minimize solvent exposure to reactive interfaces. While conventional approaches rely on cathode-electrolyte interphase (CEI) stabilization, solvation structure itself can kinetically regulate oxidative stability, offering an alternative perspective beyond thermodynamic considerations. Li et al. demonstrated that AGG-enriched solvation structures spatially control interfacial reactivity by reducing direct solvent exposure at the cathode interface under high voltage conditions [125]. In this study, dipropyl ether (DPE), a non-fluorinated monodentate ether with low solvation power, was employed to promote AGG formation, leading to altered Li^+^ solvation dynamics upon charging. As Li⁺ is released from the cathode, preferential coordination with anions over DPE induces a redistribution of solvation environments. This spatial separation effectively excludes solvent molecules from the cathode interface, thereby minimizing solvent oxidation. Moreover, the anion-dominated solvation structures alter the decomposition pathway, favoring anion decomposition over free solvents, which facilitates the formation of an anion-derived CEI. These findings suggest that high-voltage stability can be kinetically controlled by modulating Li^+^ solvation structures, shifting the focus from conventional CEI engineering to dynamic solvation modulation. Meanwhile, Cui et al. introduced a molecular anchoring diluent electrolyte (MADE) as an alternative approach to mitigate the interfacial reactivity of free ether solvents in dilute electrolytes [126]. Rather than relying on Li^+^ coordination to suppress solvent reactivity, MADE leverages strong hydrogen bonding between the diluent 1,1,2,2-tetrafluoroethyl-2,2,3,3-tetrafluoropropylether (TTE) and the ether solvent DME. This interaction weakens the binding affinity between DME and Li^+^, enabling CIP formation, while the highly diluted salt concentration (~ 0.19 M) shifts the dominant solvation structure in MADEs toward DME–TTE complexes, in contrast to the Li^+^–DME and DME–FSI^–^ complexes prevalent in LHCEs. Molecular dynamics (MD) and density functional theory (DFT) calculations confirmed that DME–TTE complexes exhibit a significantly higher oxidation potential than DME–FSI^–^ complexes, leading to improved oxidation stability. Furthermore, TTE actively participates in SEI and CEI formation, introducing both fluorinated and organic species into interfacial layers. Unlike anion decomposition, which primarily generates LiF, TTE decomposition provides a balanced composition, enhancing interfacial stability while maintaining flexibility to accommodate volume changes during Li plating and stripping.

#### Beyond CIP & AGG: contrasting ion clustering strategies

The solvation structures of electrolytes in lithium metal batteries have been categorized into solvent-separated ion pairs (SSIPs), contact ion pairs (CIPs), and aggregates (AGGs). While these classifications have provided a foundation for electrolyte design, recent studies suggest that solvation structures extend beyond these conventional models, offering new pathways for tuning lithium-ion transport, charge transfer, and interfacial stability. Among these emerging strategies, two fundamentally distinct approaches—compact ion-pair aggregate (CIPA) electrolytes and high entropy electrolytes (HEEs)—have demonstrated how mesoscale solvation engineering can enhance lithium metal battery performance. While CIPA electrolytes promote the formation of large, densely packed ion aggregates, optimizing interfacial electron transfer and SEI formation, HEEs disrupt ion clustering through solvation disorder, enhancing Li^+^ mobility and charge transfer kinetics. Despite their contrasting mechanisms, both approaches share a common goal: stabilizing the lithium metal interphase.

**Fig. 10:**
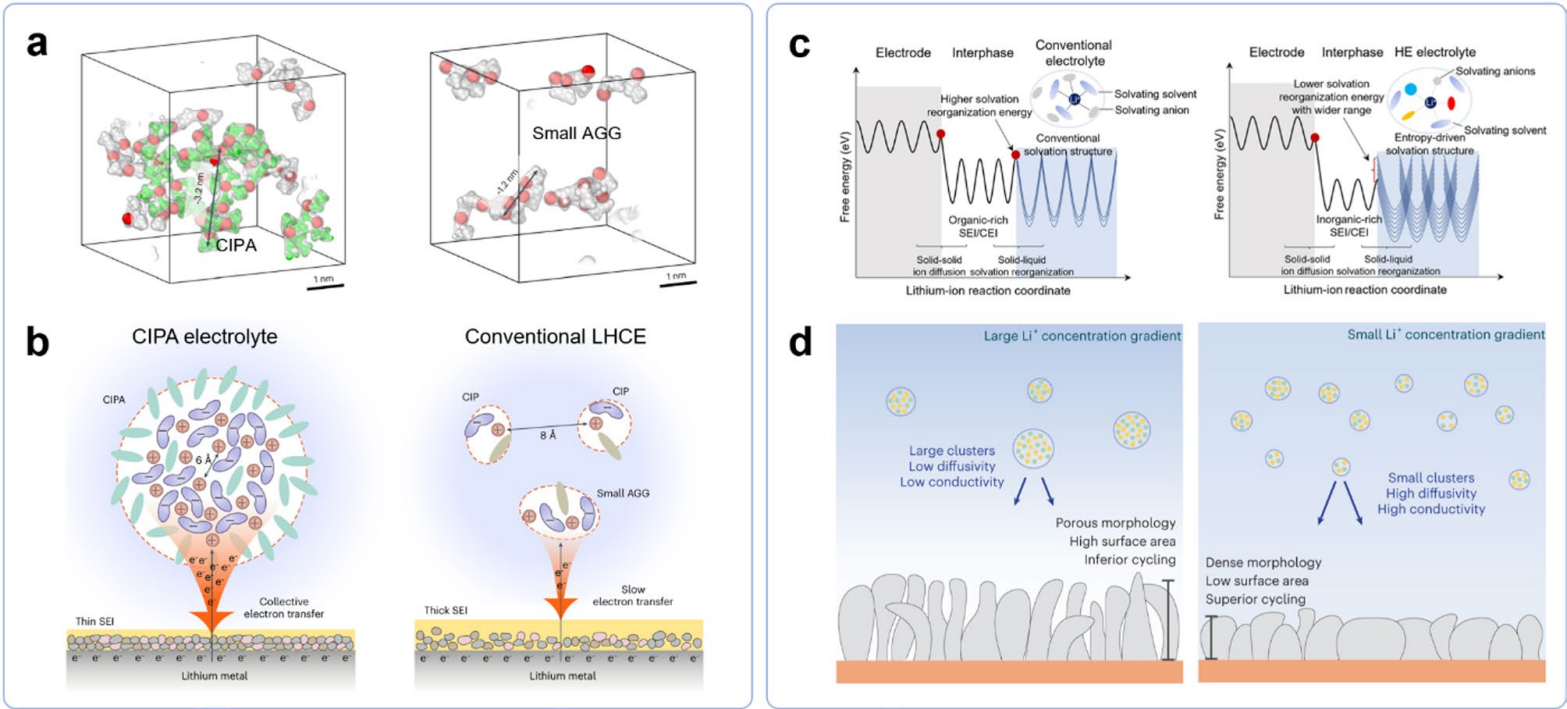
**a**) MD snapshots of AGG structures in CIPA (left) and conventional LHCEs (right). **b**) Schematic of solvation structures and interfacial reaction mechanisms in the CIPA electrolyte and conventional LHCEs. Reproduced with permission from Ref. [130]. Copyright 2024, Springer Nature. **c**) Illustration of ion transport at the electrode/electrolyte interface in terms of solvation reorganization energies. Reproduced with permission from Ref. [131], CC BY 4.0. **d**) Schematic correlation between solvation structures and lithium deposition morphology. Reproduced with permission from Ref. [132]. Copyright 2023, Springer Nature

Recent findings have shown that solvation structures are not limited to well-defined ion pairs and small aggregates, but can instead form larger, nanometric aggregates (n-AGGs) in high-concentration electrolytes [127] and multivalent-ion systems [128]. Compared to conventional AGGs, n-AGGs form extended ionic domains ( > ~ 1 nm) composed of tens to hundreds of ions, leading to distinct bulk electrolyte structuring and interfacial kinetics [129]. In line with this, Jie at al. introduced the CIPA electrolyte in lithium metal battery systems, demonstrating how densely packed solvation structures can enhance interfacial stability and electrochemical performance [130]. CIPA electrolytes achieve this unique solvation structure through the selective use of ethylene glycol di-n-butyl ether (EGBA), which bridges multiple Li^+^ ions in a trans configuration by donating one oxygen atom to each, effectively linking adjacent Li^+^ centers and compacting the solvation structure. This specific solvation arrangement promotes an FSI^–^ anion-dominated primary solvation sheath, facilitating the self-assembly of ion pairs into large-sized CIPAs. Compared to conventional LHCEs, which contain small AGGs (~ 1 nm) with longer Li^+^–Li^+^ distances (~ 8 Å), CIPA electrolytes exhibit large aggregates (3–4 nm) with reduced Li⁺–Li⁺ distances (~ 6 Å) (Fig. 10a). This structural compaction enhances interfacial anion reduction kinetics through a collective electron-transfer mechanism, accelerating the formation of a highly stable, low-organic-content SEI (Fig. 10b). As a result, CIPA electrolytes enabled 500 Wh kg^–^^1^ lithium metal pouch cell performance under lean electrolyte conditions (E/C ≈ 1.25 g Ah^–^^1^), highlighting the effectiveness of mesoscale solvation structuring in stabilizing lithium metal interfaces.

While CIPA electrolytes amplify ion clustering to enhance interfacial electron transfer, HEEs take the opposite approach, leveraging solvation disorder to disrupt clustering. Wang et al. developed a high entropy electrolyte by incorporating multiple lithium salts (LiFSI, LiTFSI, LiDFOB, and LiNO_3_) in a dilute electrolyte [131]. The inclusion of multiple anionic species increases solvation diversity, leading to a broader distribution of Li^+^ coordination environments. Spectroscopic and simulation results suggest that this diversity weakens the interactions between lithium ions and surrounding solvents or anions, which in turn enhances lithium ion diffusivity and ionic conductivity. Notably, although Li^+^–anion interactions are weaker under low-concentration conditions, a larger fraction of anions still participate in SEI formation. This counterintuitive effect leads to the development of an inorganic-rich interphase that enhances interfacial stability. Furthermore, the greater diversity in solvation structures broadens the range of solvation energies, lowering reorganization energy barriers and facilitating lithium-ion diffusion as well as charge transfer at the electrode/electrolyte interface (Fig. 10c). HEEs also mitigate the solubility limitations of key salts, which have remained underutilized despite their known benefits in SEI formation due to poor solubility in conventional solvents [133]. By increasing entropy of mixing, HEEs lower the Gibbs free energy of dissolution, enabling the incorporation of salts that would otherwise precipitate or remain inactive. This added flexibility in electrolyte composition further enhances interfacial stability and electrochemical performance.

Expanding the concept of HEEs, Kim et al. demonstrated that increasing solvation entropy through multiple solvents in LHCEs allows anion-rich solvation structures to be retained while reducing ion clustering [132]. At the microscopic level, anion-rich solvation structures were preserved, whereas mesoscopic ion clustering at the nanometer scale was reduced, as characterized by synchrotron-based X-ray scattering and molecular dynamics simulations. In most liquid electrolytes, enthalpic forces favor clustering whereas entropy favors dissociation. Thus, HEEs with high solvation entropy will lead to more dissociated electrolytes with smaller ion clusters. Electrolytes with smaller ion clusters exhibit higher diffusivity, which in turn enhances ionic conductivity by facilitating more efficient ion transport (Fig. 10d). Consequently, HEEs with smaller, more mobile ion clusters facilitate enhanced ion transport, improving performance in high-rate and high-power battery applications.

### SEI-forming additives

Electrolyte additives offer a targeted approach to stabilizing lithium deposition and regulating interfacial chemistry. Even in trace amounts, well-engineered additives can dramatically enhance battery performance by influencing Li^+^ transport, modifying interphase composition, and improving passivation without requiring significant changes to the bulk electrolyte. Through mechanisms such as selective adsorption and electrostatic shielding, these additives regulate interfacial reactions, ensuring stable cycling [134]. Some reinforce SEI structure while remaining electrochemically inert, whereas others extend their function beyond the anode and simultaneously stabilize both the anode and cathode to mitigate crosstalk effects.

**Fig. 11:**
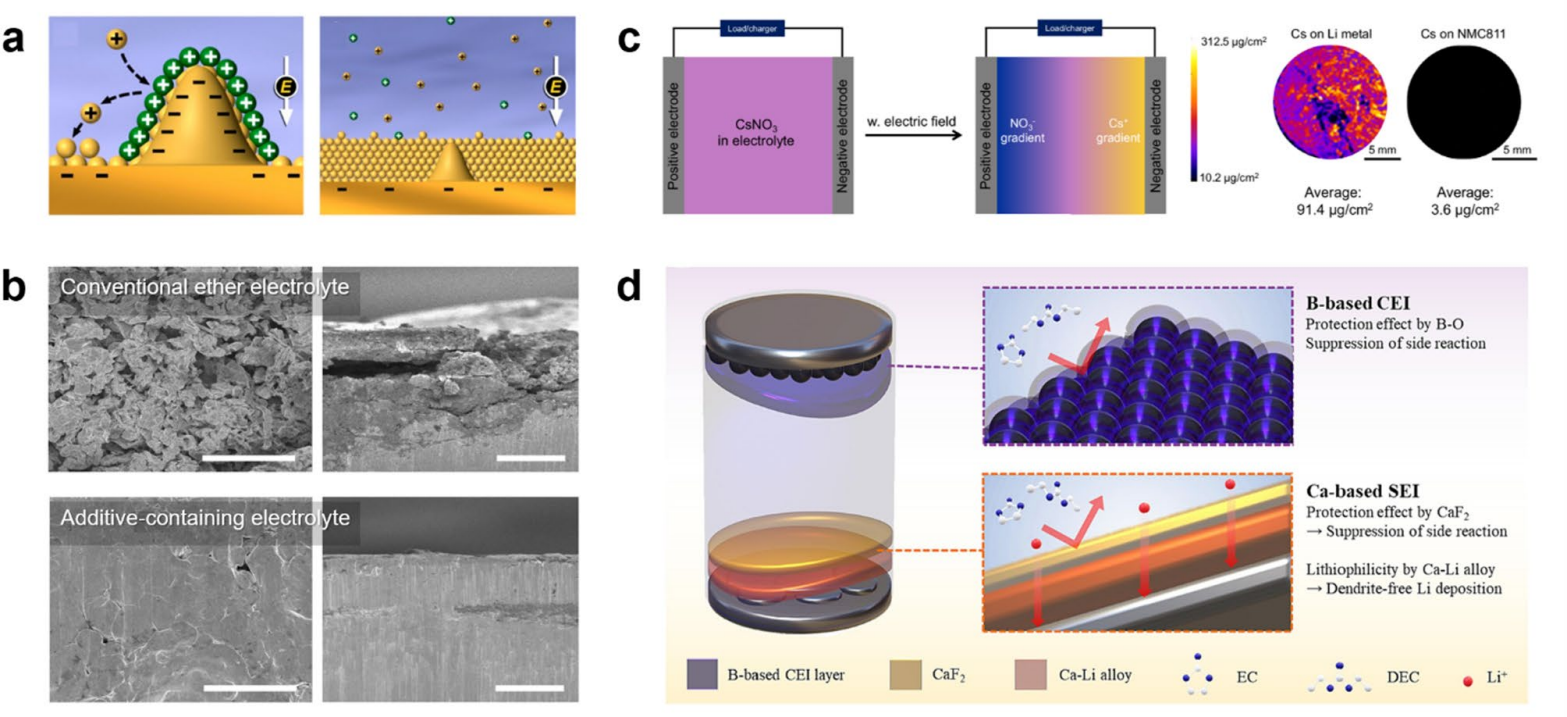
**a**) Schematic representation of the electrostatic shielding effect. Reproduced with permission from Ref. [135]. Copyright 2013, American Chemical Society. **b**) SEM images of LMAs cycled in conventional ether electrolyte (upper panel) and additive-containing electrolyte (lower panel) under 1 mA cm^–2^ and 1 mAh cm^–2^ for 100 cycles. Reproduced with permission from Ref. [137], CC BY 3.0. **c**) Illustration of the concentration gradient development of the Cs^+^ and NO_3_^−^ species under an applied electric field. Reproduced with permission from Ref. [138], CC BY 4.0. **d**) Schematic of the working mechanism of Ca(BF_4_)_2_ electrolyte additive, highlighting its distinct benefits for the cathode and anode. Reproduced with permission from Ref. [139]. Copyright 2024, Elsevier

Electrostatic shielding has been widely recognized as an effective mechanism for regulating Li^+^ flux, promoting uniform deposition, and suppressing dendrite growth (Fig. 11a). Early studies introduced alkali metal cations such as Cs^+^ and Rb^+^ as electrolyte additives to leverage this effect [135]. Due to their lower reduction potentials than Li^+^, these cations do not undergo electroplating but instead accumulate around protrusions, redirecting Li^+^ deposition away from the tip and promoting a smoother deposition layer. While this approach successfully influenced Li growth morphologies, its effectiveness was primarily limited to physical charge redistribution. Building upon this foundational concept, later studies refined the strategy by incorporating anion selection to further optimize SEI composition and stability. Wang et al. introduced potassium nonafluoro-1-butanesulfonate (KPBS), which integrates electrostatic shielding with SEI engineering [136]. While K^+^ modulates Li^+^ flux, PBS^–^ anions participate in SEI formation, promoting a LiF-rich interphase that reinforces passivation and mitigates electrolyte decomposition. Unlike Cs^+^ and Rb^+^, K^+^ has a higher standard reduction potential (–2.931 V vs. SHE) than Li^+^ (–3.040 V vs. SHE), yet its effective reduction potential in the electrolyte remains lower due to ion concentration effects, preventing electroplating and allowing K^+^ to persist as a field regulator guiding uniform Li deposition. Similarly, Ryu et al. demonstrated that bulky organic cations can regulate lithium deposition while preserving electrolyte conductivity [137]. Instead of an alkali metal cation, tetrabutylammonium tetrafluoroborate (TBATFB) utilizes bulky TBA^+^ cations that accumulate at dendrite tips, shielding Li^+^ flux and suppressing uncontrolled growth, while the TFB^–^ anion fluorinates the lithium surface, forming a robust interphase (Fig. 11b).

While these electrolyte additives primarily focus on stabilizing lithium deposition, long-term cycling stability also requires mitigating cathode degradation. Crosstalk between the anode and cathode accelerates interfacial instability, underscoring the need for simultaneous regulation of reactions at both electrodes. Rahman et al. demonstrated that cesium nitrate (CsNO_3_) extends beyond electrostatic shielding to influence interphase composition at both electrodes (Fig. 11c) [138]. Rather than simply accumulating at dendrite sites, Cs^+^ integrates into the SEI, forming cesium bis(fluorosulfonyl)imide (CsFSI)—a unique inorganic species that enhances interfacial stability while remaining free of LiF. Unlike conventional SEI components such as Li_2_O or LiF, CsFSI does not necessarily require electron consumption during its formation, potentially improving Coulombic efficiency. Meanwhile, NO_3_^–^ decomposition at the cathode mitigates transition metal dissolution, reducing the risk of metal deposition on the lithium surface and ensuring synchronized stabilization across both electrodes. Kim et al. introduced calcium tetrafluoroborate (Ca(BF₄)₂) as a dual-functional additive that stabilizes both the anode and cathode interfaces [139]. At the anode, Ca²⁺ reduces to form a Li-Ca alloy, enhancing interfacial lithiophilicity and suppressing dendrite growth. At the cathode, BF₄⁻ oxidation generates a boron-based protective layer that mitigates structural deterioration and transition metal leaching. By ensuring interfacial stability at both electrodes, this additive approach mitigates crosstalk effects and extends cycle life under demanding conditions (Fig. 11d).

### Surface modification approaches

While electrolyte engineering has been extensively explored to optimize SEI properties, direct surface modification of lithium metal anodes offers more precise control over the interfacial properties. Electrolyte modifications continuously reinforce the SEI during cycling, yet often lead to inhomogeneous and unstable layers due to complex electrochemical reactions [140–142]. Moreover, electrolyte modifications affect both the anode and cathode interfaces, sometimes triggering undesirable side reactions at the cathode that degrade overall battery performance. This section examines surface modification strategies for lithium metal anodes focusing on three key mechanisms: enhancing mechanical robustness, improving ion transport within the SEI, and promoting lateral diffusion at the lithium/SEI interface. These approaches offer tailored solutions to address the challenges at the lithium anode interface with greater precision.

#### Enhancing mechanical robustness for structural stability

**Fig. 12:**
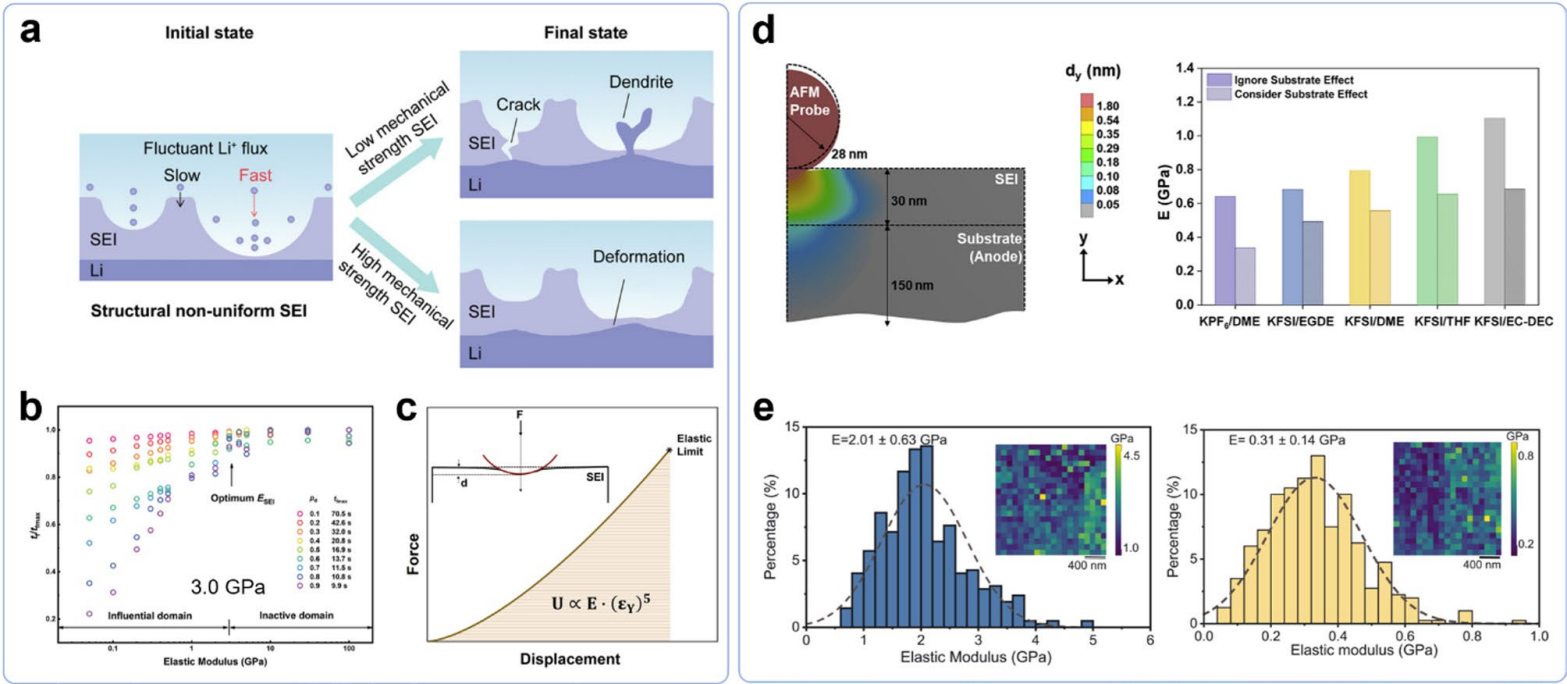
**a**) Schematic illustration of the detrimental effect of nonuniform SEI on lithium deposition. **b**) SEI failure time as a function of elastic modulus of SEI (*E*_SEI_) and SEI defect depth ratio (*p*_d_), where *t*_fmax_ denotes the maximum failure time for each *p*_d_. Reproduced with permission from Ref. [143]. Copyright 2020, John Wiley & Sons, Inc. **c**) Force-displacement curve illustrating SEI mechanical behavior, with the shaded region representing the maximum elastic deformation energy (*U*). **d**) Deformation distribution of an SEI-anode system during indentation (left) and Young’s modulus of SEI with and without substrate interference correction (right). Reproduced with permission from Ref. [144]. Copyright 2021, Elsevier. **e**) Histograms of the elastic modulus for wet-SEI and of dry-SEI. Reproduced with permission from Ref. [65]. Copyright 2022, The American Association for the Advancement of Science

The mechanical properties of the SEI play a crucial role in maintaining its structural integrity during lithium plating and stripping processes. Both theoretical and experimental studies have shown that SEI uniformity and mechanical strength significantly influence its stability and effectiveness in suppressing dendrite formation. Shen et al. demonstrated through theoretical modeling that structural uniformity governs ionic flux distribution across the SEI layer [143]. Defects in the SEI structure create localized hotspots for Li^+^ flux, accelerating lithium deposition and forming lithium protuberances that generate local stress, ultimately leading to SEI deformation and failure (Fig. 12a). Notably, an elastic modulus of approximately 3.0 GPa has been identified as sufficient for SEI stability, suggesting that extremely high modulus values are unnecessary for practical applications (Fig. 12b).

Beyond conventional mechanical assessments based on Young’s modulus, Gao et al. introduced maximum elastic deformation energy (*U*) as a more comprehensive indicator of SEI stability [144]. This parameter, which incorporates Young’s modulus and elastic strain limit, exhibits a strong correlation with Coulombic efficiency, whereas individual mechanical properties show no such correlation (Fig. 12c). A higher *U* value enables the SEI to absorb more energy through elastic deformation without mechanical failure, significantly improving cycling performance.

While atomic force microscopy (AFM)-based nanoindentation is commonly used to evaluate SEI mechanical properties, several factors must be carefully considered. Even at shallow indentation depths, the deformed region often extends beyond the SEI to the underlying substrate, potentially leading to an overestimation of Young’s modulus (Fig. 12d) [144]. Additionally, the mechanical properties of the SEI in an operational cell environment, where it is wetted by the electrolyte, differ substantially from dry state measurements. Electrolyte-induced swelling softens organic SEI components, reducing the elastic modulus by an order of magnitude (Fig. 12e) [65]. Understanding these factors is essential for obtaining meaningful insight into SEI mechanical behavior under practical cell conditions.

#### Native passivation layer modification

The intrinsic reactivity of lithium metal results in the spontaneous formation of a native passivation layer during storage, even under controlled environments. This layer, primarily composed of Li₂CO₃, LiOH, and Li₂O, exhibits non-uniform chemical composition, leading to heterogeneous electrochemical kinetics [145, 146]. Additionally, lithium oxide components in the native passivation layer impose substantially higher resistance to lithium ion diffusion compared to the SEI formed through electrochemical reactions with the electrolyte, promoting uneven lithium deposition [146]. Once lithium breaches the more vulnerable regions of the native oxide layer, subsequent deposition preferentially occurs at these sites rather than forming new pathways.

**Fig. 13:**
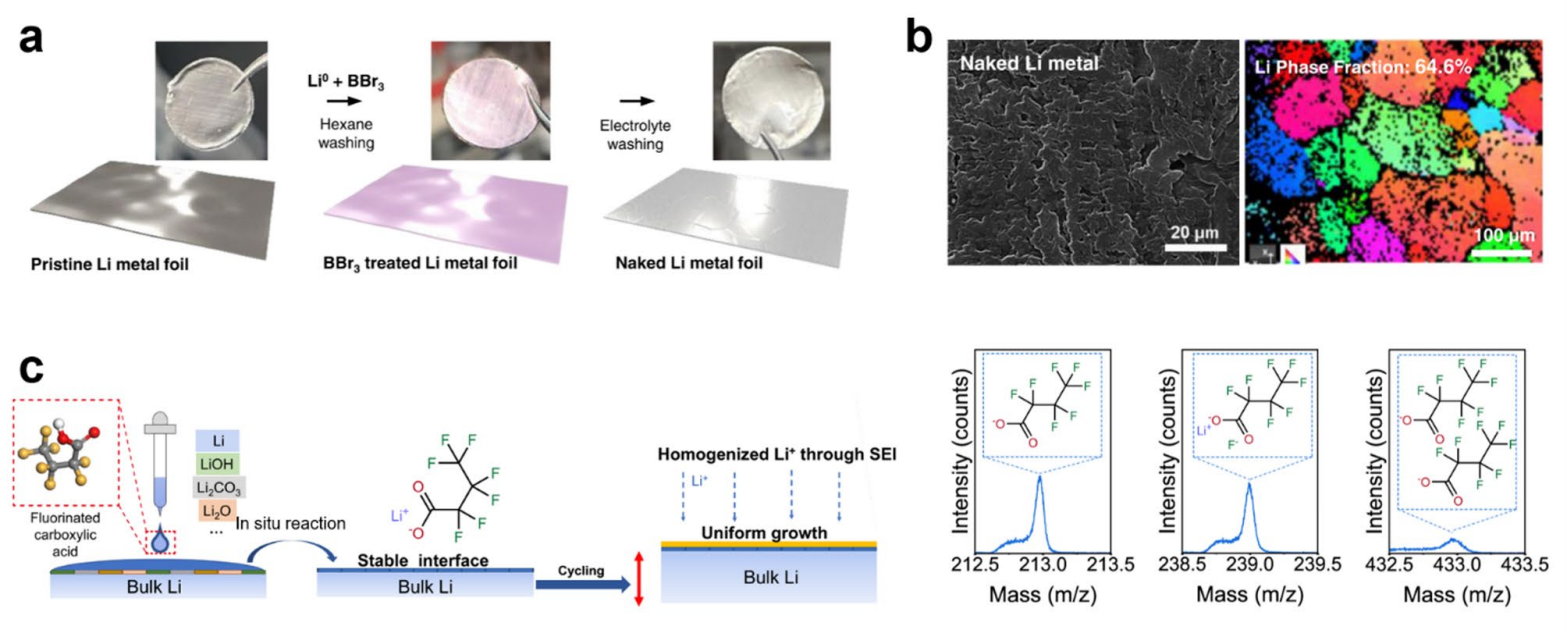
**a**) Schematic of native film removal via BBr_3_ treatment. **b**) SEM and EBSD images of naked Li metal foils after galvanostatic electrodeposition at 1 mA cm^− 2^ with 1 mAh cm^− 2^. Reproduced with permission from Ref. [146], CC BY 4.0. **c**) Schematic of HFA surface treatment (left) and TOF-SIMS characterization of HFA-treated lithium (right). Reproduced with permission from Ref. [145], CC BY 4.0

To overcome these challenges, various strategies have been developed. Baek et al. utilized a bromine-based acid-base reaction to chemically remove Li_2_O, temporarily replacing it with LiBr, which was later washed away by the electrolyte before cell assembly, yielding a passivation-free lithium surface (Fig. 13a) [146]. By eliminating the native passivation layer, this method enabled homo-epitaxial lithium plating, leading to improved cycling stability (Fig. 13b). Similarly, Sun et al. employed fluorinated carboxylic acid, specifically heptafluorobutyrate acid (HFA), to eliminate the native passivation layer and construct a lithium fluorocarbon-containing surface layer (Fig. 13c) [145]. This modified surface promoted uniform lithium deposition, thereby enhancing interfacial uniformity and long-term stability.

#### Enhancing ion transport within SEIs

**Fig. 14:**
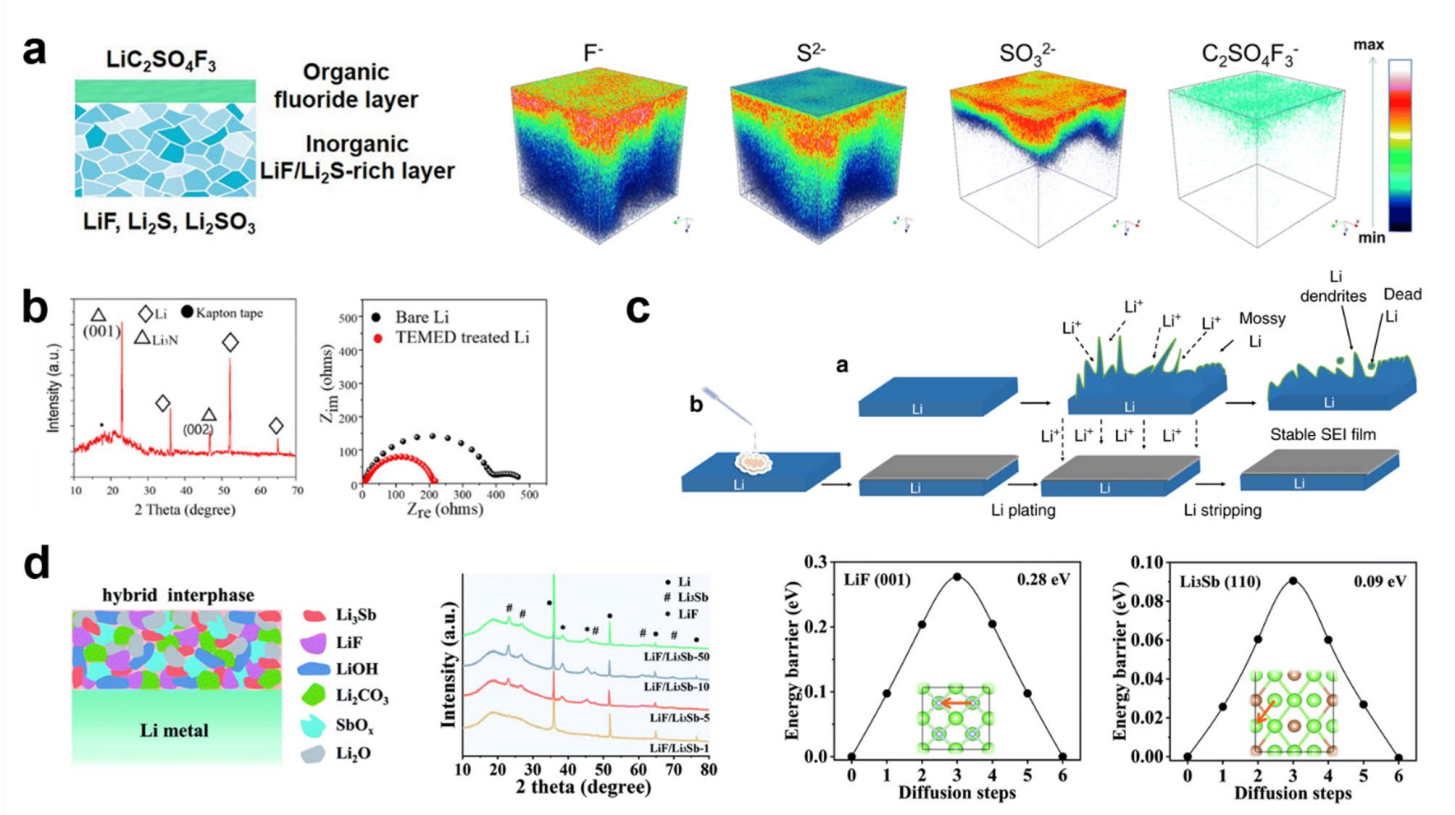
**a**) In situ formation of organic fluoride layer and inorganic LiF/Li_2_S/Li_2_SO_3_ layers. Reproduced with permission from Ref. [147]. Copyright 2024, Royal Society of Chemistry. **b**) XRD and EIS measurements of TEMED-treated lithium. Reproduced with permission from Ref. [148], CC BY 4.0. **c**) Schematic of lithium deposition behavior on bare Li and hybrid SEI-protected Li. Reproduced with permission from Ref. [149], CC BY 4.0. **d**) Construction and characterization of an artificial hybrid interphase. Reproduced with permission from Ref. [150], CC BY-NC 3.0

Efficient lithium-ion diffusion within the SEI is essential for minimizing concentration polarization, a key factor in dendrite suppression. Multi-component SEIs have emerged as promising strategies to enhance ion transport. Pang et al. reported that a gradient multi-component SEI formed through the spontaneous in-situ reaction between lithium metal and 2,2-difluoro-2-(fluorosulfonyl) acetic acid (DFFSA) [147]. This reaction eliminated the uneven native oxide layer and created a structured SEI consisting of an organic fluorinated carboxylate outer layer and an inorganic inner layer composed of LiF, Li₂S, and Li₂SO₃ (Fig. 14a). The organic outer layer enhanced wettability between the lithium metal and electrolyte, effectively reducing interfacial impedance, while the gradient inorganic inner layer facilitated lithium-ion diffusion, reduced polarization, and promoted uniform lithium deposition.

The crystalline orientation of SEI components also impacts ion transport. Pokharel et al. demonstrated that highly oriented α-phase Li₃N, synthesized via tetramethylethylenediamine (TEMED) treatment, significantly facilitated lithium-ion transport [148]. Compared to a polycrystalline Li_3_N SEI formed via conventional methods, this orientation lowered the Li^+^ diffusion energy barrier, leading to decreased concentration gradients and more uniform lithium electrodeposition (Fig. 14b).

Ion-conductive alloys provide a promising approach to enhance ion transport within SEIs. Liang et al. demonstrated that lithium-rich ion-conductive alloys, coupled with an electronically insulating surface component, effectively prevent dendrite growth [151]. Through the direct reduction of metal chlorides by lithium, a protective film comprising lithium-rich Li_x_M alloys (M = In, Zn, Bi, or As) and insulating LiCl is formed. Unlike conventional alloy anodes, which serve solely as lithium reservoirs, this approach utilizes the underlying lithium foil as the active lithium source while benefiting from the fast lithium conduction of Li_x_M alloys. These alloys exhibit higher lithium diffusion coefficients than lithium metal, effectively suppressing lithium dendrite formation. Meanwhile, the insulating LiCl component inhibits lithium reduction on the surface, generating a driving force for lithium deposition beneath the protective layer.

Expanding on this strategy, later studies incorporated LiF as an insulating component due to its superior properties. LiF has been widely recognized for its excellent electronic insulating properties and mechanical strength [149, 150]. However, the high ion migration barrier of LiF limits the diffusion kinetics of lithium ions through the SEI, resulting in large lithium plating/stripping overpotentials, especially at high rates [150]. To overcome this limitation, hybrid SEI strategies have emerged that incorporate lithium alloy components with high diffusion properties alongside LiF. The synergistic effect of electronic insulation properties of LiF and the lithium alloy acting as an ionic channel enables fast lithium-ion diffusion and uniform lithium deposition at the SEI/lithium interface. Pathak et al. fabricated a fluorinated hybrid SEI composed of LiF and a Sn–Li alloy phase using a replacement reaction between lithium metal and SnF₂ (Fig. 14c) [149]. This artificial SEI not only ensured fast lithium-ion diffusion and suppressed lithium dendrite growth but also provided a synergistic effect by storing lithium via reversible Sn–Li alloy formation while enabling lithium plating underneath it [152]. Similarly, Hu et al. constructed an artificial hybrid interphase layer using antimony trifluoride (SbF₃), which underwent a displacement reaction with lithium metal to form LiF and Li₃Sb (Fig. 14d) [150]. Zheng et al. coated CaF₂ nanoparticles onto lithium foil, leading to the spontaneous formation of LiF and a Li–Ca alloy on the lithium surface [153]. The CaF₂ coating effectively isolated lithium from the electrolyte, preventing side reactions at the interface and resulting in an LiF-dominated SEI layer, which, in combination with uniform lithium-ion distribution via the Li–Ca alloy, constructed a homogeneous and dense lithium deposition. Zhuang et al. formed a LiF-rich SEI layer doped with a Li-Au alloy by coating the separator with gold (Au), which spontaneously reacted with the lithium metal anode [154]. The resulting Li-Au alloy, with its distinctive electron-donating properties, facilitated the reductive decomposition of LiTFSI in the electrolyte, thereby generating a robust SEI layer that promoted rapid Li^+^ transport and effectively mitigated dendritic growth.

#### Promoting lateral diffusion at lithium-SEI interfaces

The interaction between the SEI and lithium metal strongly influences lithium deposition morphology. Organic SEI components typically form strong bonds with lithium, restricting diffusion along the Li/SEI interface and promoting vertical lithium penetration through the SEI, leading to dendrite formation [155]. In contrast, inorganic lithium compounds such as LiF, Li₂O, and Li₃N exhibit weak bonding and high interfacial energy, promoting lateral lithium diffusion while effectively blocking lithium penetration into the inorganic SEI. Additionally, the high Young’s modulus provides greater mechanical resistance to dendritic growth, further enhancing interfacial stability.

**Fig. 15:**
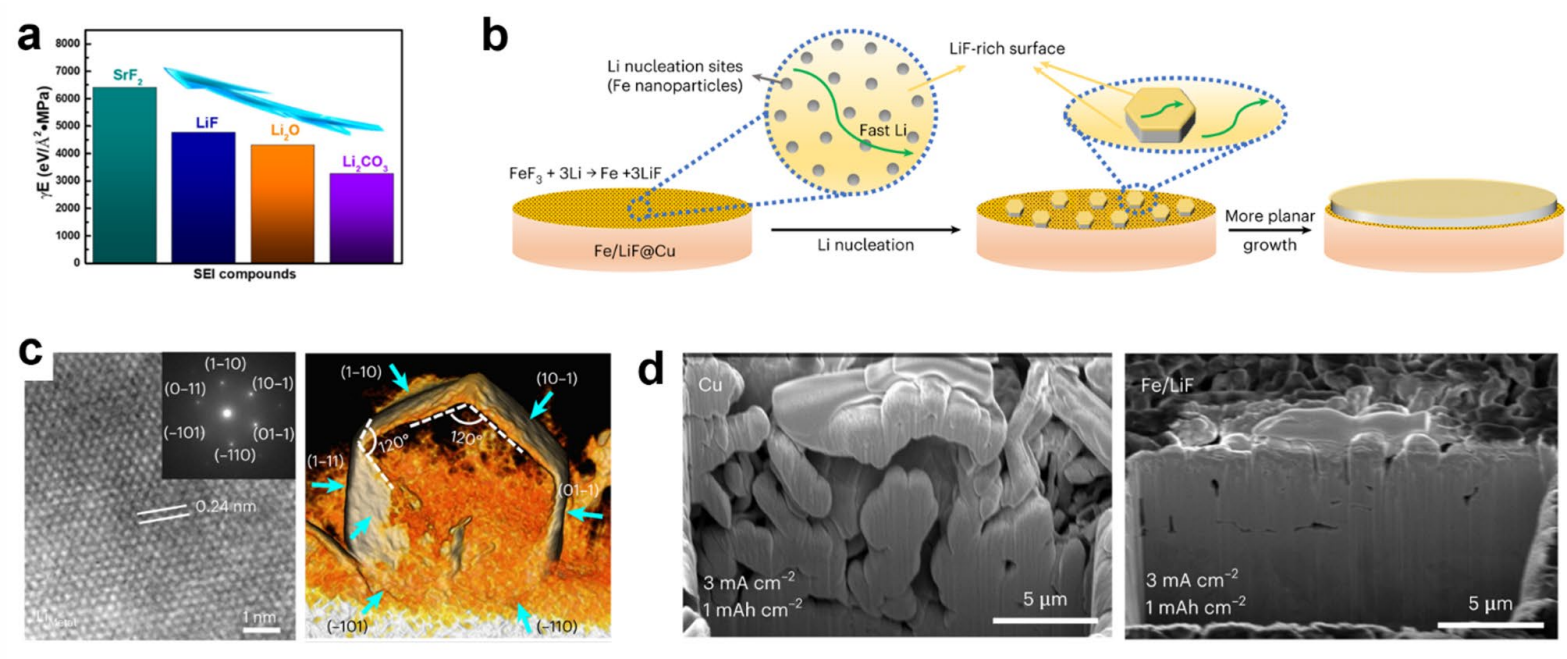
**a**) *γE* values for different SEI compounds. Reproduced with permission from Ref. [157]. Copyright 2020, American Chemical Society. **b**) Schematic representation of Li nucleation and growth on the Fe/LiF nanocomposite. **c**) Cryo-TEM imaging and crystallographic analysis of hexagonal-shaped single-crystalline lithium metal. **d**) Cross-sectional morphology of deposited Li on a Cu substrate (left) and Fe/LiF nanocomposite (right) under 3 mA cm^− 2^ and 1 mAh cm^− 2^ condition. Reproduced with permission from Ref. [158]. Copyright 2023, Springer Nature

Liu et al. proposed that a combination of interfacial energy (*γ*), which represents the energy required to form a new Li/SEI interface [156], and the Young’s modulus (*E*) of SEI governs lithium dendrite formation and growth [157]. They introduced *γE* as a criterion for evaluating lithium dendrite suppression capability. Theoretical calculations identified that LiF possesses the highest *γE* value among common SEI components, validating its effectiveness. However, LiF alone proved insufficient to fully suppress lithium dendrite formation under demanding operating conditions. DFT calculations identified SrF₂ as having an even higher *γE* value than other metal fluorides, and demonstrated its effectiveness in inhibiting dendrites when incorporated into SEIs via lithium alloying strategies (Fig. 15a).

For high-energy-density lithium metal batteries, achieving practical anode-free configurations is imperative. This requires precise control over lithium nucleation and initial growth on foreign substrates to ensure lithium metal reversibility. Thus, beyond fast lithium diffusion at the lithium/SEI interface, optimizing the Li/substrate interface is equally critical for achieving dense and uniform lithium deposition [158–161]. Wu et al. developed an FeF₃-coated Cu substrate that, upon lithiation, transformed into a uniform Fe/LiF nanocomposite (Fig. 15b) [158]. The nanosized Fe particles provided evenly distributed nucleation sites, ensuring uniform lithium seeding during the initial deposition stage, while the LiF promoted rapid lithium diffusion, guiding lithium growth toward its thermodynamically stable morphology [83]. The resulting hexagonally shaped lithium single-crystalline seeds induced dense, low-porosity lithium deposition in subsequent cycles (Fig. 15c and d).

## Considerations for expanding from lab-scale coin cells to industrial standards

Lithium metal batteries have long been studied at the lab-scale, primarily in coin cell configurations, where experimental conditions often deviate significantly from the practical requirements of high-energy applications [162, 163]. Many studies utilize coin cells under idealized conditions, such as excessive electrolyte volume and low areal capacities, which can obscure degradation mechanisms. However, as LMB research moves toward commercial viability, there is growing emphasis on adopting pouch cell configurations that better reflect the constraints encountered in industrial applications. Pouch cells impose practical limitations, such as high cathode loading, lean electrolyte conditions (low E/C ratio), and a well-defined N/P ratio, all of which significantly impact long-term stability and performance [162, 164]. As a result, promising strategies demonstrated in coin cells do not guarantee to translate effectively to practical high-energy pouch cells [162]. Moreover, among various practical cell formats—including cylindrical and prismatic types—pouch cells are particularly favored in research environments due to their flexible form factor. Unlike rigid-cell formats that require complex manufacturing processes, pouch cells are easier to assemble and modify in the lab. This flexibility enables systematic control over electrode size, thickness, and applied pressure, which is essential for controlled testing under near-practical conditions. Therefore, pouch cells serve as an effective bridge between idealized coin cell tests and commercial-scale batteries, and have become a preferred platform for assessing the translatability of lab-scale strategies.

**Fig. 16:**
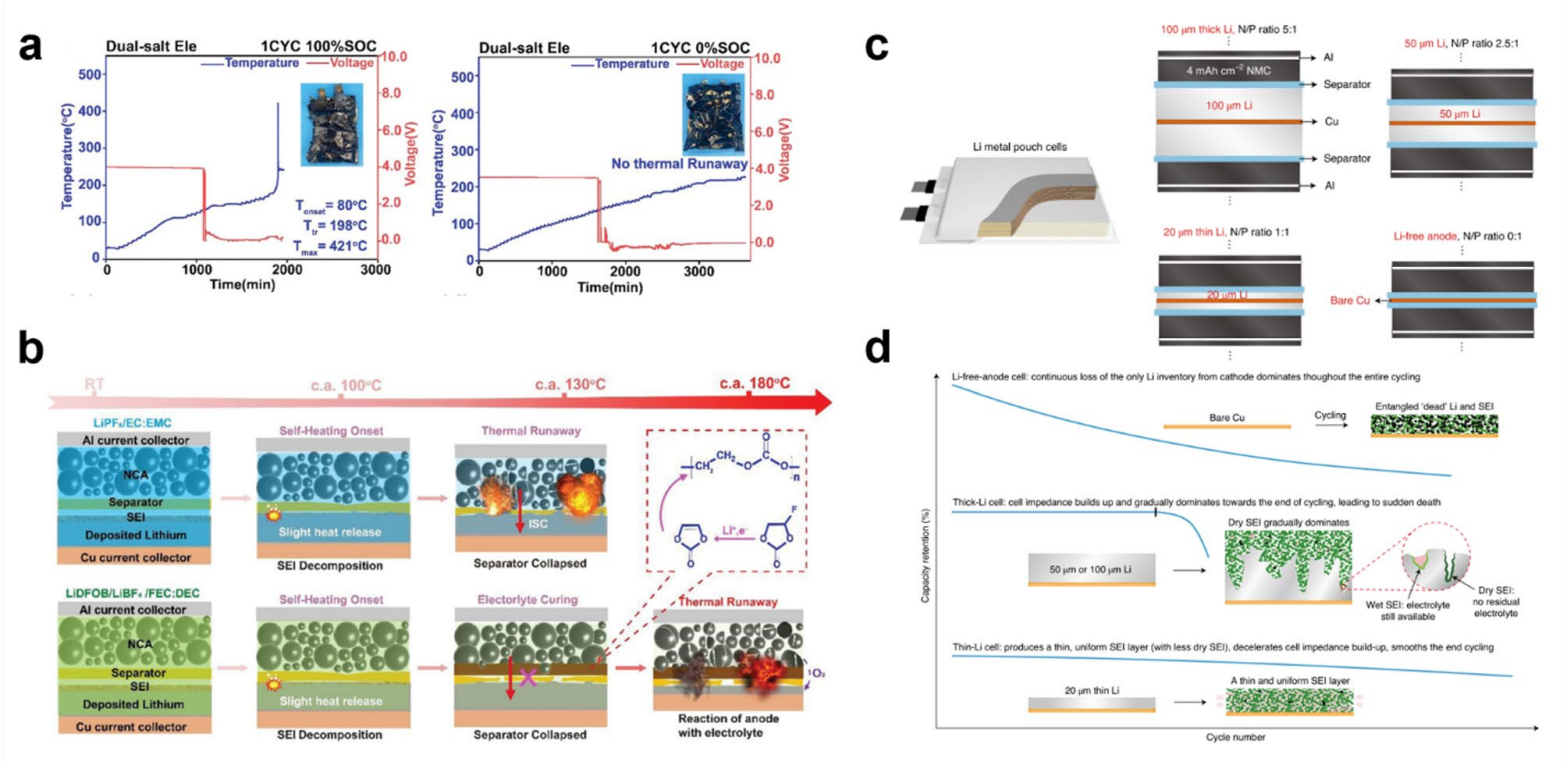
**a**) Thermal stability variations based on lithium content. **b**) Schematic of thermal runaway pathways and the role of FEC in enhancing thermal stability. Reproduced with permission from Ref. [166]. Copyright 2023, John Wiley & Sons, Inc. **c**) Schematic of Li metal pouch cells with different Li metal thicknesses. **d**) Illustration comparing degradation mechanisms in cells with varying Li metal thicknesses. Reproduced with permission from Ref. [164]. Copyright 2021, Springer Nature

As researchers shift toward pouch cell studies, one of the most critical considerations is lithium reservoir control. One proposed strategy to simplify lithium management and improve energy density is the adoption of anode-free configurations. In anode-free LMBs, lithium is not pre-deposited on the negative electrode; instead, lithium is supplied entirely from the cathode [165]. While this approach maximizes energy density and simplifies manufacturing, the absence of a lithium reservoir means that any irreversible lithium consumption—such as electrolyte decomposition—directly reduces capacity, making long-term cycle stability a major challenge. Moreover, despite the assumption that eliminating lithium metal improves safety, the risk of thermal runaway persists [166]. Anode-free lithium metal batteries are not entirely free from metallic lithium, as lithium plating occurs during charging, leading to the formation of both active and inactive lithium, which forms complicated SEI. Consequently, some of the safety concerns associated with LMBs remain relevant to anode-free designs. Zhang et al. demonstrated that under elevated temperatures, a discharged anode-free pouch cell remains stable, whereas a fully charged one undergoes thermal runaway, albeit with lower intensity than conventional LMBs (Fig. 16a) [166]. Moreover, the severity of thermal runaway was found to be closely related to the total amount of metallic lithium, while the self-heating onset temperature is determined by the chemical composition and thermal stability of the SEI layer. Notably, electrolyte formulations containing fluoroethylene carbonate (FEC) were shown to form a thermally stable insulating coating layer on the cathode surface, delaying internal short circuits and mitigating exothermic reactions (Fig. 16b). These findings indicate that electrolyte selection and interfacial stability play critical roles in determining the viability of anode-free LMBs.

Beyond anode-free configurations, recent studies suggest that a completely lithium-free anode may not always be the most effective strategy. Niu et al. systematically investigated the influence of the N/P ratio on the cycling performance of high-energy lithium metal pouch cells (Fig. 16c), challenging the conventional assumption that a thicker Li anode inherently extends cycle life [164]. By varying the lithium thickness from 100 μm to 0 μm (anode-free configuration), the study revealed that cell degradation is governed by the interplay between Li availability, electrolyte depletion, and SEI accumulation (Fig. 16d)​. While thick lithium layers (N/*P* ≥ 2.5) initially provide high CE by compensating for lithium losses at the cathode, prolonged cycling leads to electrolyte depletion and the formation of thick, porous SEI layers with limited ion conduction, ultimately increasing polarization and causing a sudden capacity drop. Conversely, in anode-free pouch cells (N/*P* = 0), the irreversible Li consumption during each cycle leads to a gradual capacity fade rather than abrupt failure. A critical finding was that an optimized N/P ratio of 1 (20 μm Li) was the most effective for balancing lithium utilization and electrolyte availability, minimizing dry SEI formation, and reducing polarization. These findings suggest that rather than defaulting to an anode-free approach, strategic Li allocation may be necessary achieve stable cycling in practical LMBs.

**Fig. 17:**
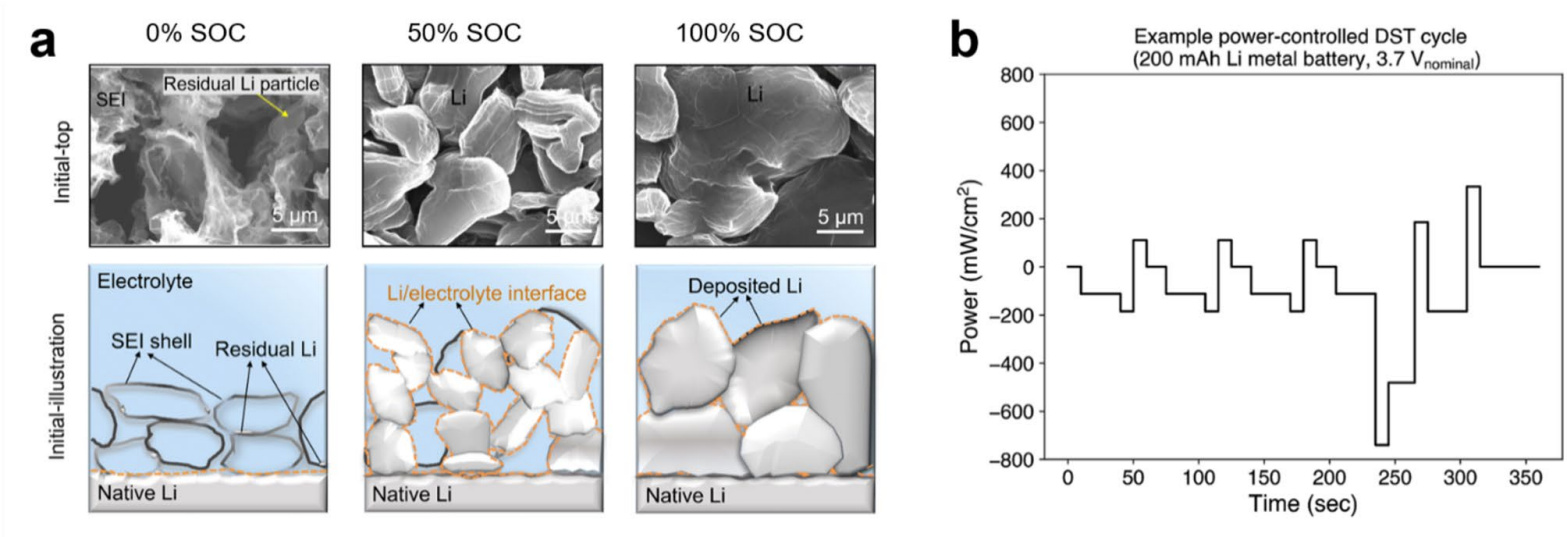
**a**) SEM images and schematic illustrations of LMAs during initial calendar aging test at different SOCs. Reproduced with permission from Ref. [19], CC BY-NC 3.0. **b**) Example of power-controlled DST cycling protocol. Reproduced with permission from Ref. [167]. Copyright 2024, Elsevier

In addition to managing lithium availability during cycling, storage stability (calendar life) is another critical factor for pouch cells. Cao et al. demonstrated that the key factor governing LMB calendar life is the surface area of lithium exposed to the electrolyte [19]​. Storing LMBs at 50% state of charge (SOC) accelerates degradation due to the formation of highly porous lithium deposits, which increase the active interface area and promote side reactions​. In contrast, storing LMBs at either 0% or 100% SOC exhibit significantly improved stability by minimizing lithium exposure (Fig. 17a). In situ TEM analysis further revealed that a mechanically robust and reusable SEI layer plays a crucial role in mitigating lithium corrosion and maintaining interfacial integrity over prolonged storage. These findings emphasize the importance of SOC management and electrolyte engineering in extending the operational lifespan of LMBs.

As LMB research moves toward industrial adoption, the need for electrochemical testing protocols that capture application-relevant conditions becomes increasingly critical. Conventional assessments primarily rely on constant-current cycling, which provides steady charge-discharge conditions that simplify analysis but fail to reflect the complex operating environments of LMB applications. For example, batteries in electric vehicles (EVs) operate under dynamic conditions with variable loads and frequent fluctuations in current demand. These factors significantly influence degradation mechanisms, including lithium plating/stripping behavior, SEI evolution, and electrolyte consumption. Recognizing this limitation, Hatzell et al. emphasized the importance of aligning laboratory-scale testing with practical performance metrics​ [167]. A power-controlled discharge testing protocol was designed to better simulate realistic driving conditions. Unlike traditional constant-current cycling, this protocol maintains constant-current charging while employing a constant-power discharge profile, which more accurately captures variations in power demands experienced during sudden acceleration and braking (Fig. 17b)​. By aligning laboratory testing methodologies with industrial deployment, this approach provides a more representative evaluation of LMB behavior under practical usage conditions.

## Conclusions

Lithium metal batteries represent a significant technological advancement beyond conventional lithium-ion batteries, offering remarkable enhancements in energy density crucial for meeting the increasing demands of energy storage applications. Despite their exceptional theoretical advantages, several fundamental challenges persist, including chemical and galvanic corrosion of lithium metal, dead lithium formation, and complexities related to the solid-electrolyte interphase. Our review systematically addressed these challenges, highlighting the insights gained from recent groundbreaking studies and summarizing current academic trends to provide clear guidance for future research directions.

Chapter 2 categorized the degradation mechanisms of lithium metal into chemical corrosion, galvanic corrosion, and dead lithium evolution, discussing their respective operating mechanisms and severity. It is identified the severity of each degradation mechanism has strong dependence on cell configuration and operating conditions. The degradation chemical corrosion is pronounced during calendar aging. Galvanic corrosion is exacerbated when copper current collector is exposed to electrolyte. The formation of dead lithium also exhibited varying degrees of severity depending on the stripping current density. Based on understanding of each degradation reaction, corresponding mitigation strategies were introduced.

Chapter 3 discussed the components and structure of the solid electrolyte interphase, providing emerging evaluations and perspectives on organic components, LiF, and Li₂O. It has been recently identified that, in lithium metal batteries, organic components undergo swelling and dissolution due to the electrolyte, negatively impacting the reversibility of the lithium metal anode. In contrast, inorganic components are considered crucial for the reversibility of the lithium metal anode, with particular attention given to LiF. LiF has become widely recognized as a key component for forming an ideal SEI in LMBs. However, recent findings have revealed new possibilities for Li₂O-based SEI, demonstrating over 99% Coulombic efficiency even in the absence of LiF. Next, the advancements in SEI analysis techniques and the improved understanding of SEI were introduced, along with the emergence of novel SEI architectures. In addition to this, research on controlling the distribution of SEI components was presented.

Chapter 4 provided a comprehensive overview of current strategic approaches to interfacial challenges, emphasizing the pivotal role of the SEI. Solvation structure engineering emerged as a central strategy for electrolyte optimization. Recent advancements highlighted the importance of precisely controlling electrolyte parameters—such as salt concentrations, solvent-to-diluent ratios, and intrinsic diluent properties—to achieve balanced performance in LHCEs. Furthermore, systematic molecular design guided by steric hindrance and electronic effects, has shown considerable promise for tuning solvation environments in WSEs. Notably, novel solvation strategies beyond conventional ion clusters (SSIP, CIP, AGG) have provided new perspective and distinct pathways for enhancing battery performance. Electrolyte additives were discussed as critical tools for controlling lithium deposition morphology and mitigating electrode crosstalk. Recent research highlighted dual-functionality additives capable of stabilizing both anode and cathode interfaces simultaneously, significantly enhancing the overall battery performance and lifespan. Beyond electrolyte modifications, direct electrode surface engineering represents another crucial approach to interfacial stability. Strategies such as optimizing native passivation layer and constructing artificial SEIs prioritize mechanical robustness, efficient ion transport within SEIs, and lateral diffusion at electrode interfaces, collectively enhancing lithium deposition uniformity and cycling reversibility.

Chapter 5 addressed the critical challenge of transitioning laboratory-scale successes into practical, commercially viable pouch-cell formats, underscoring the necessity of realistic testing protocols closely aligned with industrial standards and real-world operational conditions.

Future research should continue to deepen the fundamental understanding of solvation dynamics and SEI formation mechanisms through sophisticated analytical and computational methods. Advancements in electrolyte and SEI engineering are pivotal for realizing stable, high-performance LMBs. By systematically addressing the key issues outlined in this review—corrosion suppression, SEI structural optimization, and targeted surface and electrolyte modifications—the commercial viability and performance robustness of lithium metal batteries can be fully achieved.

## Data Availability

No new data were generated in this study. All relevant information has been obtained from published sources, which are cited in the manuscript.
